# Lifespan extension with preservation of hippocampal function in aged system x_c_^−^-deficient male mice

**DOI:** 10.1038/s41380-022-01470-5

**Published:** 2022-02-18

**Authors:** Lise Verbruggen, Gamze Ates, Olaya Lara, Jolien De Munck, Agnès Villers, Laura De Pauw, Sigrid Ottestad-Hansen, Sho Kobayashi, Pauline Beckers, Pauline Janssen, Hideyo Sato, Yun Zhou, Emmanuel Hermans, Rose Njemini, Lutgarde Arckens, Niels C. Danbolt, Dimitri De Bundel, Joeri L. Aerts, Kurt Barbé, Benoit Guillaume, Laurence Ris, Eduard Bentea, Ann Massie

**Affiliations:** 1 Laboratory of Neuro-Aging & Viro-Immunotherapy, Center for Neurosciences (C4N), Vrije Universiteit Brussel (VUB), Brussels, Belgium; 2 Department of Neurosciences, Université de Mons (UMONS), Mons, Belgium; 3 Neurotransporter Group, Department of Molecular Medicine, Institute of Basic Medical Sciences, University of Oslo, Oslo, Norway; 4 Department of Food, Life and Environmental Science, Faculty of Agriculture, Yamagata University, Yamagata, Japan; 5 Institute of Neuroscience, Université Catholique de Louvain, Brussels, Belgium; 6 Department of Medical Technology, Niigata University, Niigata, Japan; 7 Frailty in Ageing research Department, VUB, Brussels, Belgium; 8 Laboratory of Neuroplasticity and Neuroproteomics, and Leuven Brain Institute (LBI), University of Leuven, Leuven, Belgium; 9 Pharmaceutical Chemistry, Drug Analysis and Drug Information, C4N, VUB, Brussels, Belgium; 10 The Biostatistics and Medical Informatics Department, VUB, Brussels, Belgium; 11 Hospital of Jolimont, La Louvière, Belgium

**Keywords:** Neuroscience, Physiology

## Abstract

The cystine/glutamate antiporter system x_c_^−^ has been identified as the major source of extracellular glutamate in several brain regions as well as a modulator of neuroinflammation, and genetic deletion of its specific subunit xCT (xCT^−/−^) is protective in mouse models for age-related neurological disorders. However, the previously observed oxidative shift in the plasma cystine/cysteine ratio of adult xCT^−/−^ mice led to the hypothesis that system x_c_^−^ deletion would negatively affect life- and healthspan. Still, till now the role of system x_c_^−^ in physiological aging remains unexplored. We therefore studied the effect of xCT deletion on the aging process of mice, with a particular focus on the immune system, hippocampal function, and cognitive aging. We observed that male xCT^−/−^ mice have an extended lifespan, despite an even more increased plasma cystine/cysteine ratio in aged compared to adult mice. This oxidative shift does not negatively impact the general health status of the mice. On the contrary, the age-related priming of the innate immune system, that manifested as increased LPS-induced cytokine levels and hypothermia in xCT^+/+^ mice, was attenuated in xCT^−/−^ mice. While this was associated with only a very moderate shift towards a more anti-inflammatory state of the aged hippocampus, we observed changes in the hippocampal metabolome that were associated with a preserved hippocampal function and the retention of hippocampus-dependent memory in male aged xCT^−/−^ mice. Targeting system x_c_^−^ is thus not only a promising strategy to prevent cognitive decline, but also to promote healthy aging.

## Introduction

The cystine/glutamate antiporter system x_c_^−^ exchanges intracellular glutamate for extracellular cystine and has been identified as modulator of both glutamatergic neurotransmission in the central nervous system (CNS) of mice [1–3] and of (neuro)inflammation [4, 5]. Accordingly, genetic deletion of the specific subunit xCT (*Slc7a11*; xCT^−/−^ mice) [6] is protective in mouse models for (age-related) neurological disorders characterized by excitotoxicity and neuroinflammation [5, 7–11]. Yet, the oxidative shift reported by Sato et al. in the plasma cystine/cysteine balance of adult xCT^−/−^ mice led to the hypothesis that xCT deletion would accelerate the aging process [6], in line with the increasing cystine/cysteine ratio that is observed over the lifespan in humans [12]. On the other hand, the propensity of system x_c_^−^ deficiency to suppress (neuro)inflammation [4, 5] would be favorable in the context of aging, since a better control of *inflammaging*-a concept characterized by an increased pro-inflammatory status and considered one of the pillars of the biology of aging- is suggested to be key in regulating longevity [13]. Moreover, with age, increased levels of pro-inflammatory factors not only promote the onset of frailty, but also the occurrence of age-related disorders, including dementia and cognitive aging [14]. These seemingly conflicting hypotheses regarding the role of system x_c_^−^ in aging warrant a more in-depth study.

One of the most impactful consequences of an increased life expectancy is cognitive aging, which is characterized by a decline in memory that is mainly dependent on proper hippocampal function and is regulated by glutamatergic neurotransmission [15]. System x_c_^−^ is expressed on astrocytes [16] and constitutes the major source of extracellular glutamate in the hippocampus of mice [7]. This glial glutamate can modulate neurotransmission by acting on extrasynaptic metabotropic glutamate receptors [1] and by regulating postsynaptic expression of AMPA receptors [3]. However, enhancement of system x_c_^−^ by e.g., inflammatory stimuli or oxidative stress [9] leads to increased glutamate release into the extracellular space. Glutamate released by astrocytes surrounding the synaptic cleft is believed to be more prone to induce excitotoxicity by acting on extrasynaptic NMDA receptors [17, 18]. Aging promotes the migration of NMDA receptors from synaptic to extrasynaptic sites and an alteration in the NMDA receptor expression has been correlated to age-related decline in spatial learning [19, 20]. Moreover, the vulnerability of the aging hippocampus to excitotoxicity is reinforced by the impairment of astrocytic glutamate uptake [21]. System x_c_^−^deficiency could therefore protect against age-related memory decline by preventing extrasynaptic NMDA receptor overactivation.

Besides its role as neurotransmitter, glutamate is associated with several metabolic processes, including the biosynthesis of nucleic acids, proteins, and metabolic intermediates such as acetyl coenzyme A, which is on its turn involved in lipid synthesis and mitochondrial metabolism. Both life- and healthspan, including cognitive function, are affected by age-related metabolic changes that lead to disrupted metabolic homeostasis and deficiencies in cell respiration [22, 23]. Maintaining the intracellular glutamate pool by inhibiting system x_c_^−^ could thus support several of the metabolic pathways impacted by aging, as has been shown in cancer cells that were deprived of glucose [24, 25].

Altogether, both direct and indirect evidence suggests that absence of system x_c_^−^ would be protective against age-related excitotoxicity and (neuro)inflammation, and has the potential to maintain proper cellular functioning during metabolic stress. Therefore, this study aims at investigating the role of system x_c_^−^ in physiological aging in mice, with a focus on the immune system and how this relates to hippocampal function and hippocampus-dependent memory. We here show that absence of system x_c_^−^ extends lifespan of male mice and protects against age-related priming of the innate immune system. Although we did not detect major dissimilarities in hippocampal inflammatory markers, several metabolic pathways were differently affected by age in the hippocampus of xCT^−/−^ mice, compared to xCT^+/+^ mice. In addition, we observed beneficial effects of xCT deletion on the age-related changes in the morphology of hippocampal CA1 neurons and in the functionality at hippocampal CA3-CA1 connections, as well as protection against hippocampus-dependent memory decline. To conclude, our results provide fundamental information about the function of system x_c_^−^ in the aging process and give novel insights into the mechanisms underlying age-related dysfunctions, and by extension healthy aging.

## Materials and methods

### Animals

Adult (3–4 months) and aged (18–24 months) male xCT^−/−^ mice and their wild-type littermates (xCT^+/+^ mice) were bred in a heterozygous colony and genotyped as described [6, 10]. These littermates were complemented with mice born from homozygous xCT^−/−^ and xCT^+/+^ breeders, to ensure sufficiently large groups of age-matched mice. These homozygous breeders were first-generation offspring of heterozygous breeding couples, to prevent genetic drift. Mice were group-housed under standardized conditions (20–24 °C, 10/14 h dark/light cycle, 45–65% humidity) with free access to water and food. Unless stated otherwise, mice were sacrificed using cervical dislocation. Experiments were approved by the Ethical Committee for Animal Experiments of the Vrije Universiteit Brussel or University of Mons and carried out according to the national guidelines on animal experimentation.

All analyses were performed by a researcher blinded for genotype and age of the mice, and all recordings and images were analyzed in a random manner. An overview of the total number of mice used as well as the distribution of the cohorts over the different experimental setups can be found in Supplementary Table 1.

### Life- and healthspan

Lifespan was evaluated by recording the date of spontaneous death. Animals showing objective signs that indicate a high probability of dying within 24–48 h [26], were euthanized and the date of euthanasia was recorded as date of death (3 xCT^+/+^ mice and 8 xCT^−/−^ mice). Median lifespan represents the time upon which only 50% of the group is still alive and the maximum lifespan is the median of the longest lived 20% of the mice in each group. For general health assessment, mice were weighed, and their body temperature was recorded using a rectal probe (RET-3, ADInstruments). The glucose tolerance test was performed by i.p. injecting 2 g/kg glucose and measuring glucose levels in blood obtained from tail snips using a blood glucose meter (ACCU-CHEK^®^ Aviva). The clinical frailty index was assessed as described previously [27]. When the mice were sacrificed, cardiac blood was collected in Li-heparin coated tubes and centrifuged (2000 × *g*, 3 min) to collect plasma; organs were isolated and their weights were normalized to the weight of the mouse. Li-plasma samples were analyzed on the automated platform Roche Cobas 6000 system (Roche Diagnosis, Germany) with c501 chemistry module (see Supplementary Methods for clinical chemistry).

### Dynapenia and sarcopenia

Dynapenia was evaluated by measuring the limb muscle strength (Grip Strength Meter; Bioseb). Mice were allowed to grasp a grid with all four of their limbs and the peak pull force in grams was recorded at the time the mouse releases its paws from the grid. The averaged value of three measurements was normalized to the body weight of the mouse. Adult mice were tested once while the aged mice were tested monthly between 18 and 20 months. Post mortem, the *musculus gastrocnemius* was dissected, weighed, and normalized to the weight of the mouse, as a measure for sarcopenia.

### Plasma concentration of cysteine, glutathione, and their oxidized forms

Blood was collected via submandibular bleeding into EDTA-containing tubes and centrifuged (3 min, 2000 × *g*, 4 °C); 20 µl plasma was transferred to a tube containing 20 µl of N-ethylmaleimide. After 10 min incubation, 40 µl of 100% methanol and 40 µl of 10 µM L-methionine sulfone (internal standard) were added and samples were incubated on ice for 2 h. The samples were centrifuged (15 min, 15,000 × *g*, 4 °C), supernatants filtered (Filter Millex-LH, 0.45 µm, Merck laboratories) and 60 µl was lyophilized using a rotavap system. The pellet was dissolved in 20 µl of 50% acetonitrile and the concentration of cystine, cysteine, glutathione (GSH) and glutathione disulfide (GSSG) were analyzed using HPLC-MS [28].

### Assessment of age-induced priming of the immune system

Mice were i.p. injected with bacterial lipopolysaccharide (LPS) (0.20 mg/kg; serotype 0111:B4, Sigma-Aldrich) or sterile saline (0.9% NaCl, B. Braun Vet Care) [29]. Body temperature was measured 2, 4, 6 and 24 h post-injection to evaluate sickness. Three aged mice of each genotype were sacrificed between 6 and 24 h after LPS injection as they reached a humane endpoint. A second cohort of mice was sacrificed 3 h after LPS administration, using an overdose of pentobarbital (Dolethal^®^, 200 mg/kg i.p., Vetoquinol). Cardiac blood was collected in Li-heparin coated tubes and centrifuged (3850 × *g*, 15 min, 4 °C). Plasma cytokine levels were analyzed with a customized Bio-Plex Pro Assay (Bio‐Rad Laboratories; multiplexed: IL-1-β (171G5002M), TNF-α (171G5023M), IL-10 (171G5009M), IL-6 (171G5007M)) and Luminex 200 system (Merck laboratories).

### Immunohistochemistry

Hemispheres were post-fixed in 4% formaldehyde (Sigma-Aldrich) for 72 h and 40 µm vibratome sections were made. Three sections containing the hippocampus (between −1.82 and −2.3 mm relative to the bregma) were stained for microglial Iba-1 or astrocytic GFAP, as described before [4] and using antibodies as detailed in Supplementary Table 2, diluted in 20% normal goat serum. Photomicrographs of the dentate gyrus were taken at 20x magnification using an Axioskop 40 microscope connected to a digital camera (AxioCam ERc 5s, Carl Zeiss Microscopy GmBH). Cell number and morphology were analyzed using Image J software and as illustrated in Supplementary Fig. 1.

### Real-time PCR

Total hippocampal RNA was extracted and reverse transcribed into cDNA as described [4]. Real-time PCR was performed using the TaqMan^®^ Universal Master Mix (Applied Biosystems) and commercial primer assays (Supplementary Table 3). Using geNorm, Ywhaz was selected as the most stable reference gene. PCR reactions for all samples were performed in duplicate, and on each plate a non-template control was included. Data were analyzed using the 2^−ΔΔCt^ method.

### Metabolomics

Untargeted metabolomics was performed by Metabolon (www.metabolon.com) on snap-frozen mouse hippocampus that was stored at −80 °C until further processing, as described [30, 31]. Sample preparation was performed using the automated MicroLab STAR^®^ system (Hamilton Company). Recovery standards were added for quality control (QC) purposes. Proteins were precipitated with methanol under vigorous shaking for 2 min (Glen Mills GenoGrinder 2000) followed by centrifugation. The resulting extract was divided into different fractions for analysis by reverse phase (RP)/UPLC-MS/MS with positive ion mode electrospray ionization (ESI), RP/UPLC-MS/MS with negative ion mode ESI and by hydrophilic interaction liquid chromatography (HILIC)/UPLC-MS/MS with negative ion mode ESI. Samples were placed briefly on a TurboVap^®^ (Zymark) to remove organic solvent. The sample extracts were stored overnight under nitrogen before preparation for analysis. Experimental samples were randomized across the platform run with QC samples and blanks spaced evenly among the injections. Raw data was extracted, peak-identified and QC processed using Metabolon’s hardware and software. Library entries of purified standards or recurrent unknown entities were used to identify compounds. Metabolon maintains a library based on authenticated standards that contains the retention time/index (RI), mass to charge ratio (m/z), and chromatographic data (including MS/MS spectral data) on all molecules present in the library. Peaks were quantified using area-under-the-curve. Principal component, random forest and pathway enrichment analysis were performed by Metabolon using ArrayStudio, R statistics and JMP software on log transformed data. For pathway enrichment only pathways with more than five metabolites detected were taken into consideration. When comparing metabolic pathways, matched measures one-way ANOVA with a Geisser-Greenhouse correction for unequal variability of differences and Tukey’s multiple comparisons test was used on log2 transformed data. To compare changes in specific metabolite levels, two-way ANOVA with Sidak’s multiple comparison test was used on scaled and imputed data (i.e., raw area counts for each biochemical are rescaled to set the median equal to 1 and missing values are imputed with the minimum). See Statistics section and Supplementary Table 4 for exceptions.

### Golgi-Cox staining

Brains were stained using the FD Rapid GolgiStain Kit (FD Neurotechnologies Inc.) and sliced into 80 µm vibratome sections. Images of 3–5 hippocampal CA1 pyramidal neurons per animal were obtained using a bright-field Zeiss Axio Imager Z.1 connected to an AxioCam MRc5 camera (Carl Zeiss Microscopy GmBH). Clearly isolated neurons with consistent staining throughout the whole neuron and without any truncated dendrites were selected. Photomicrographs were taken with a 100× oil-objective and z-stacks (0.5 μm/stack) were used to image the whole neuron. The number of primary dendrites was counted and the complexity of the basal and apical tree was analyzed by Sholl analysis. Spine density was calculated on 3–4 10 µm segments per neuron. These segments were apical second- or third-order dendritic branches located at a distance of at least 100 µm from the soma (mid-apical regions of the *stratum radiatum*). A 2D tracing of the neurons was made for Sholl analysis, using the Neuromantic software; all other analyses were performed directly on the 100× magnified z-stacks in Image J-win64 (Fiji). The mean of all neurons was calculated for each animal and considered as *n* = 1.

### Electrophysiology

The electrophysiology setup was prepared and hippocampal sections were processed as detailed before [32]. Following a resting period of 90 min, a bipolar nickel–chromium stimulating electrode was used to stimulate the Schaffer collateral fibers, and a glass microelectrode (2–5 MΩ, filled with aCSF) was positioned in the *stratum radiatum* of CA1 to record the field excitatory postsynaptic potentials (fEPSPs). By gradually increasing the stimulation intensity (from 2 to 10 V), an input/output (I/O) curve was generated. Next, fEPSPs were recorded at 40% of the maximum amplitude obtained in the I/O experiment. When a stable baseline (32 min) was obtained, long-term potentiation (LTP) was induced by applying a single train of high-frequency stimulation (100 Hz, 1 s) [33]. After induction of LTP, fEPSPs were recorded for 4 h. Data acquisition and analysis were performed using WinLTP. For each slice, the fEPSP slopes were normalized to the average slope over the baseline. The ability of the neurons to induce and maintain LTP was evaluated by the ratio of the normalized slope 4 h after LTP induction to the one before LTP induction as well as to the one immediately after LTP induction.

### Barnes maze

Mice were trained twice a day with an inter-trial interval of 20 min, for 5 consecutive days. Primary escape latency and distance (i.e., the time required and the distance traveled before the mouse locates the escape hole) were measured using an automated video tracking system (Ethovision, Noldus). Mice were trained until all groups had an equal performance when considering the primary distance, a parameter that is not biased by reduced speed of aged mice. To evaluate short- and long- term memory, a test trial of 90 s was performed respectively 24 h and 6 days after the last training session. Mice that were unable to locate the escape hole, received a score of 90 s for the primary latency; mice that did not move were excluded for the primary distance and for the search strategy. The used search strategy was analyzed manually and scored as direct (going directly to the escape hole and making three or fewer mistakes), serial (visiting consecutive holes in a serial manner before finding the escape hole), random (random crossings of the platform before visiting the escape hole) or serial/random [34].

### Statistics

Data are presented as mean ± SEM. Statistical analyses were performed using GraphPad Prism 8–9 software or SPSS version 25. Two groups were compared using a Mann–Whitney test; four groups with two variables were compared using a two-way ANOVA followed by Sidak’s multiple comparisons test. The survival curve was analyzed using a Log-rank (Mantel-Cox) test and for categorical data a Chi-square test was used. The α-value was set at 0.05. For parametric analyses, the normality of the residuals was assessed using the D’Agostino and Pearson omnibus normality test, and the Browne-Forsythe test was used to test for equal variances. Data not showing a normal distribution, were transformed before applying the parametric two-way ANOVA. However, for the ease of interpretation of the data, we show the graphs with the non-transformed data for all experiments. The data not showing a normal distribution after transformation, were analyzed using a non-parametric one-way ANOVA (on the non-transformed data). An outlier test (Grubbs’ test) was performed when a data point was out of the range of the data points of all groups. Only significant outliers that were identified as described above (α-value set at 0.05), have been removed from the dataset. All details on statistical analyses as well as outlier detection, are given in Supplementary Table 4. Details about the longitudinal analyses used for analyzing the I/O curves of the slice electrophysiology experiment and the Sholl analysis data of the Golgi-Cox experiment, are described in Supplementary Methods. Details with regards to the statistical analysis of the metabolomics data are discussed in the respective section. As we are the first to report the effects of xCT deletion on healthy aging, prior reports of expected effect size could not be used as a guide for sample size estimation. For the analyses reported, the experimental design was therefore based on typical sample size values from previous studies using similar techniques.

## Results

### xCT deletion extends lifespan without major effects on general health parameters

**Fig. 1:**
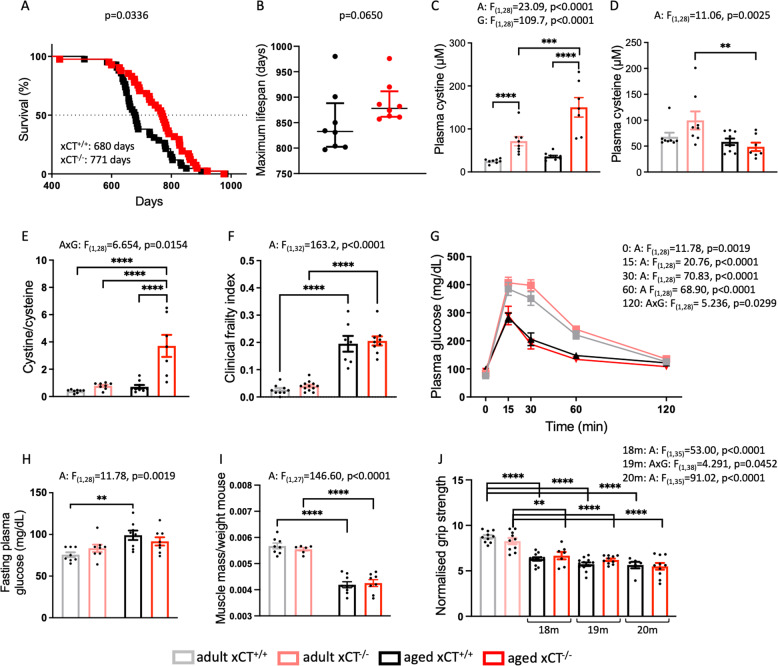
Despite the induction of an oxidative shift in the plasma cystine/cysteine redox couple of aged mice, absence of xCT increases lifespan without affecting general health parameters. Lifespan of xCT^+/+^ and xCT^−/−^ mice was determined using a Kaplan-Meier curve and analyzed with a Log-rank test (**A**, *n* = 41–42 mice/group). To evaluate the maximum lifespan, the lifespan of the longest lived 20% of the mice in each group was plotted as median ± interquartile range (**B**, Mann–Whitney test, *n* = 8 mice/group). Plasma concentrations of cystine (**C**) and cysteine (**D**) were used to calculate the cystine/cysteine ratio (**E**, *n* = 7–10 mice/group). Age-related clinical deterioration was studied by the clinical frailty test (**F**, *n* = 7–12 mice/group), the glucose tolerance test (**G**) and the fasting glucose levels (**H**, *n* = 8 mice/group). Sarcopenia was evaluated by weighing the *musculus gastrocnemius* (**I**, *n* = 6–9 mice/group) and dynapenia was analyzed by measuring the grip strength of the mice (**J**, *n* = 6–12 mice/group). Data are presented as mean ± SEM and analyzed using a two-way ANOVA followed by Sidak’s multiple comparisons test (see Supplementary Table 4). For the whole limb force measurements, this test was performed for each of the respective ages, compared to the adult mice. **p < 0.01; ***p < 0.001; ****p < 0.0001. Significant main effects are presented in the figure: A aging effect, G genotype effect, AxG interaction effect.

In our survival study, xCT deletion resulted in a prolonged median lifespan (Fig. 1A) and a strong trend for an increased maximum lifespan (Fig. 1B), despite an increased cystine/cysteine ratio in the plasma of aged xCT^−/−^ mice, compared to age-matched xCT^+/+^ mice and adult mice (Fig. 1C–E). The redox potential (E_h_) for the cystine/cysteine couple was calculated using the Nernst equation and showed an oxidative shift of 8.5 mV between adult and aged xCT^+/+^ mice, corresponding to the linear increase of 0.16 mV per year in humans [12]. This shift is, however, increased to 28 mV in the plasma of aged xCT^−/−^ mice.

To investigate if the beneficial effects on longevity are perhaps the result of reduced reductive stress [35], characterized by a buildup of reducing equivalents, we measured other relevant redox markers. No age- or genotype-related changes were detected in the plasma GSSG/GSH ratio (Supplementary Fig. 2A–C). The lactate/pyruvate ratio, commonly used as a marker for the NADH/NAD^+^ status [36], was increased with age, independent of the genotype (Supplementary Fig. 2D–F).

The oxidative shift in the cystine/cysteine redox couple of aged xCT^−/−^ mice did also not negatively impact the general health status of the mice. The clinical frailty index increased with age, independent of the genotype (Fig. 1F). Interestingly, no difference in clinical frailty index was observed between both genotypes when the mice approached their maximum lifespan (i.e., 22–24 months for xCT^+/+^ mice and 24–28 months for xCT^−/−^ mice; Supplementary Fig. 2G). In line with previous studies in C57BL/6J mice [37], we observed increased glucose tolerance with aging, independent of the genotype (Fig. 1G). The age-related increase in fasting glucose levels was, however, less pronounced in xCT^−/−^ compared to xCT^+/+^ mice (Fig. 1H). Furthermore, xCT deletion had no effect on age-related sarcopenia and dynapenia (Fig. 1I, J). Accordingly, analysis of general health parameters such as body weight and temperature as well as weight of different organs, revealed similar age-related changes in xCT^+/+^ and xCT^−/−^ mice (Supplementary Fig. 2H–N). Also, blood chemistry showed aging effects that were mainly independent of the genotype of the mice (Supplementary Fig. 3).

### xCT deletion attenuates age-related priming of the innate immune system

**Fig. 2:**
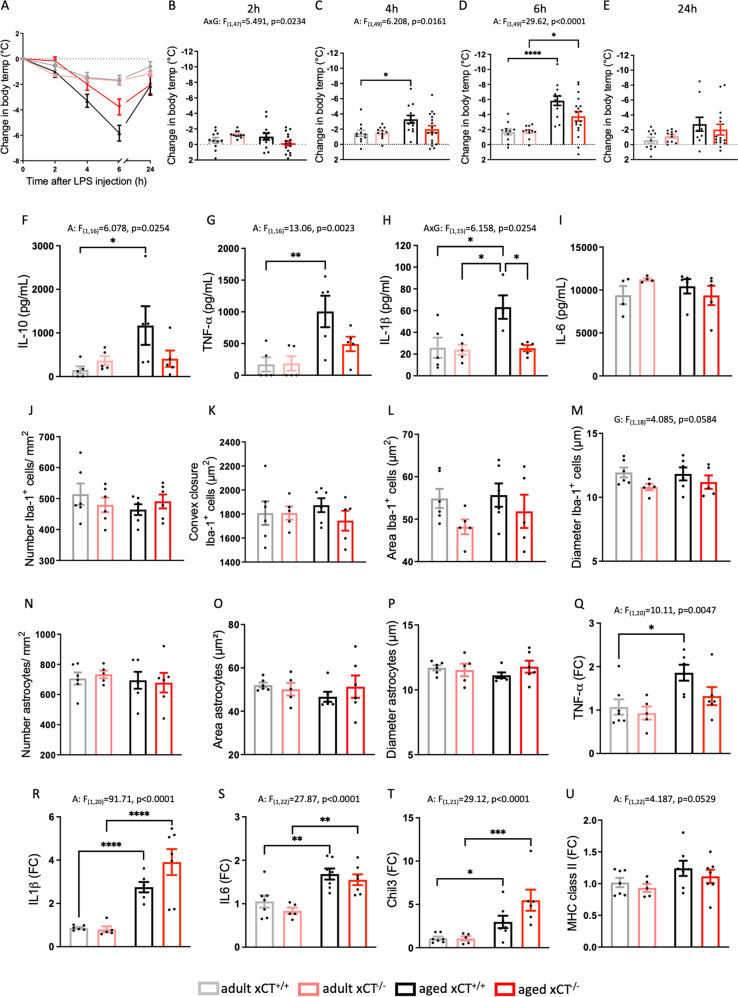
Absence of xCT attenuates age-induced priming of the peripheral innate immune system with only minor effects on the age-related pro-inflammatory shift in the hippocampus. Priming was evaluated by comparing the reaction of adult (*n* = 11 mice/group) and aged (*n* = 13–18 mice/group) xCT^+/+^ and xCT^−/−^ mice to an i.p. LPS injection (0.20 mg/kg) (**A**–**I**). LPS-induced hypothermia was followed over time (**A**–**E**, changes in body temperature are plotted relative to the baseline). The plasma concentrations of IL-10 (**F**), TNF-α (**G**), IL-1β (**H**), and IL-6 (**I**) were measured 3 h post-injection (*n* = 4–5 mice/group). Microglial activation and astrogliosis were studied in healthy adult and aged hippocampus of xCT^−/−^ and xCT^+/+^ mice, by quantifying the number and morphology of Iba-1^+^ (**J**–**M**) and GFAP^+^ cells (**N**–**P**), at the level of the dentate gyrus. Data represent the mean value of 2–3 sections per mouse (*n* = 6 mice/group). Representative pictures are shown in Supplementary Fig. 1 and demonstrate the area defined as the convex closure. Using real-time PCR for TNF-α (**Q**), IL-1β (**R**), IL-6 (**S**), and chitinase-like protein-3 precursor (Chil3, **T**) the hippocampal inflammatory environment was analyzed (*n* = 4–7 mice/group). Finally, MHC class II mRNA levels were studied as a marker for priming of the central immune system (**U**). Data are presented as mean ± SEM and analyzed using a two-way ANOVA followed by Sidak’s multiple comparisons test, or a Kruskal–Wallis test in case of non-normal distributed data (**E**) (see Supplementary Table 4): *p < 0.05; **p < 0.01; ***p < 0.001; ****p < 0.0001. Significant main effects are presented in the figure: A aging effect, G genotype effect, AxG interaction effect.

In the periphery, system x_c_^−^ is expressed on cells of the innate immune system and on activated lymphocytes [9, 38]. The function of system x_c_^−^ on immune cells, however, remains understudied, and the effect of xCT deletion on age-related changes in the immune system, has never been addressed. We here show that absence of system x_c_^−^ did neither alter the splenic population of T cells and innate immune cells, nor the typical age-induced changes in these populations (Supplementary Figs. 4 and 5). Besides T-cell senescence, age-induced priming of the innate immune system is a well-described feature of inflammaging [39]. In aged mice, a primed immune system will show an enhanced response to a low dose of LPS [40]. LPS injection induced hypothermia in all groups of mice (Fig. 2A), with an age-induced enhancement that was mainly driven by the xCT^+/+^ mice (Fig. 2B–E). Three hours after LPS injection, plasma levels of both pro-inflammatory (TNF-α, IL-1β) and anti-inflammatory (IL-10) cytokines were significantly higher in aged mice, an effect that is again mainly supported by the xCT^+/+^ mice (Fig. 2F–H). No effect was seen in the plasma levels of IL-6 (Fig. 2I). Taken together, the attenuation of the enhanced reactivity of aged xCT^−/−^ mice to LPS, supports reduced inflammaging in the absence of system x_c_^−^.

### Absence of system x_c_^−^ alters the hippocampal metabolome, with only minor effects on hippocampal inflammation in aged mice

Immune-CNS communication can underlie -or participate in- neurological dysfunction in pathological or age-related conditions [41]. System x_c_^−^ was reported to modulate both systemic and central inflammation [4, 5], and xCT expression can be induced by typical hallmarks of aging, i.e., inflammation and oxidative stress [9]. Hippocampal xCT protein expression (Supplementary Fig. 6G) and cellular distribution (Supplementary Fig. 6A–F) remained, however, unaltered with age. Moreover, the activity of system x_c_^−^ was unaffected in the aged hippocampus (Supplementary Fig. 6H).

We further studied whether genetic xCT deletion and the associated reduced priming of the peripheral immune system impacts the inflammatory status of the hippocampus, in physiological conditions. Aging had no effect on either the number of microglia and astrocytes, or on their morphological characteristics (Fig. 2J–P). However, xCT deletion did induce an overall decrease in the diameter of Iba-1^+^ cells, suggesting less reactive microglia (Fig. 2M). Hippocampal mRNA levels of pro-inflammatory cytokines were increased in aged mice (Fig. 2Q–S), and for TNF-α this was mainly driven by the xCT^+/+^ mice (Fig. 2Q). Aging induced an increase in both the anti-inflammatory chitinase-like protein-3 precursor (Chil3) (Fig. 2T) and the MHC class II expression, a marker for priming, independent of genotype (Fig. 2U). Altogether, these data suggest that absence of xCT slightly shifts the inflammatory environment in the aged hippocampus towards a more anti-inflammatory state.

**Fig. 3:**
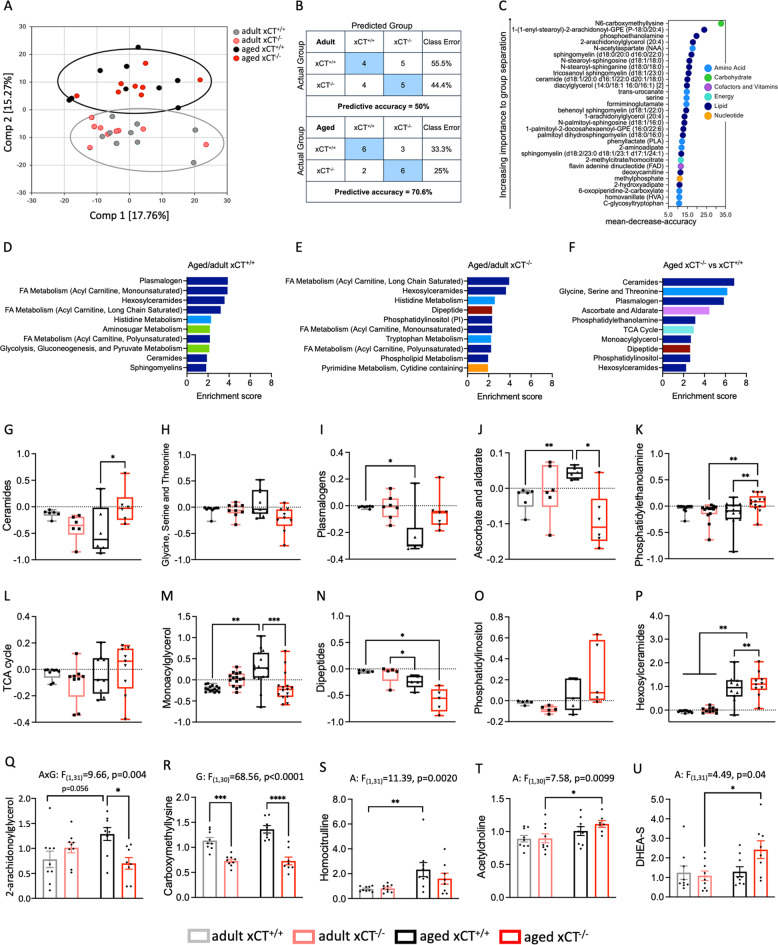
Absence of xCT affects the hippocampal metabolome in aged but not in adult mice. Principal component analysis (**A**) and random forest analysis (confusion matrix (**B**) and biochemical importance plot (**C**)) were performed on the hippocampal metabolome (*n* = 8–9 mice/group). Pathway enrichment analysis was applied to identify relevant pathways altered in aged xCT^+/+^ (**D**) and xCT^−/−^ mice (**E**), compared to their adult controls. The pathways identified as different between aged xCT^−/−^ compared to aged xCT^+/+^ mice (**F**), were further analyzed in more detail (**G**–**P**). Results are expressed as log2 fold changes (each dot represents the average of a single metabolite within a group). Statistical analysis was performed using a matched measures one-way ANOVA with a Geisser-Greenhouse correction for unequal variability of differences and Tukey’s multiple comparisons test (**G**, **H**, **J**, **L**, **N**–**P**) or a Friedman test followed by multiple comparisons with Dunn’s correction in case of non-normal distributed data (**I**, **K**, **M**). Metabolites not identified by pathway enrichment analysis due to rigor default settings (only pathways with more than five metabolites are considered), were selected based on relevance and statistical significance (two-way ANOVA with Sidak’s multiple comparison test) and are represented as mean scaled and imputed data ± SEM (**Q**–**U**) (see Supplementary Table 4). A aging effect, G genotype effect, AxG interaction effect; *p < 0.05; **p < 0.01; ***p < 0.001; ****p < 0.0001, DHEA-S sulfate ester of dehydroepiandrosterone, FA fatty acid, TCA tricarboxylic acid.

Systemic oxidative or reductive stress as well as inflammation -as seen in aging and being affected by xCT deletion- can have profound effects on brain metabolism [42, 43]. Moreover, system x_c_^−^ deficiency can also directly affect cellular metabolism [24, 25]. To investigate if and how hippocampal metabolism is differentially affected by aging in the absence of xCT, we performed untargeted metabolomics on samples of aged and adult xCT^−/−^ and xCT^+/+^ mice. The hippocampal metabolome of adult mice was minimally affected in the absence of xCT (Fig. 3A, B). However, random forest classification could assign 70.6% of the aged mice to the correct genotype, implying a dissimilar metabolome between both genotypes at an older age (Fig. 3B). The biochemical importance plot mainly showed differences in lipid and amino acid metabolism (Fig. 3C). Pathway enrichment analysis revealed that in xCT^+/+^ mice aging mainly induced changes in different lipid subclasses (Fig. 3D). In aged xCT^−/−^ hippocampus, similar pathways were affected; however, whereas the group of plasmalogens are highly disrupted with age in xCT^+/+^ mice (Fig. 3D), it is not present in the top ten of affected pathways in the xCT^−/−^ mice (Fig. 3E). Moreover, a comparison of the metabolome of the aged xCT^+/+^ and xCT^−/−^ hippocampus showed key differences in several groups of glycerolipids, sphingolipids, and one-carbon metabolism (Fig. 3F). When plotting the altered metabolic groups separately and including the adult mice in the comparison (Fig. 3G–P), a profound effect was seen in the group of plasmalogens (Fig. 3I), vitamin C metabolism (Fig. 3J) and monoacylglycerols (Fig. 3M), in which absence of xCT prevented the age-related changes observed in the xCT^+/+^ mice. Especially the levels of the monoacylglycerol 2-arachidonoylglycerol were significantly lower in the aged xCT^−/−^ compared to aged xCT^+/+^ hippocampus (Fig. 3Q).

Hippocampal levels of N6-carboxymethyllysine, the single most important metabolite for group separation by random forest analysis (Fig. 3C), were significantly lower in xCT^−/−^ mice, compared to age-matched xCT^+/+^ controls (Fig. 3R). Homocitrulline showed an overall aging effect, with a significant increase in the aged compared to the adult xCT^+/+^ hippocampus (Fig. 3S). The neurotransmitter acetylcholine and the sulfate ester of the hormone dehydroepiandrosterone (DHEA-S) were significantly increased with aging; an effect that is mainly supported by the xCT^−/−^ mice (Fig. 3T, U).

Comparable to the observations made in the plasma, the lactate/pyruvate ratio showed a borderline significant increase in the aged hippocampus of both genotypes (Supplementary Fig. 7E–G). Finally, no differences could be observed in total tissue GSH, GSSG and cysteine levels (Supplementary Fig. 7A–D). The latter finding is in accordance with the unaltered GSH content as well as the absence of overt signs of oxidative stress that we previously reported in the hippocampus of adult and aged xCT^−/−^ mice [7], confirming earlier findings that system x_c_^−^ is not critical for the synthesis of GSH in vivo and alternative pathways are active to maintain the (intracellular) levels of cysteine and GSH [44]. To conclude, several of the metabolic changes that we observed in the aged xCT^−/−^ mice could be beneficial to hippocampal function in the aged brain.

### Absence of system x_c_^−^ alters the morphology of CA1 neurons and prevents age-related changes in hippocampal function

As the main source of hippocampal glutamate [7], and modulator of both neuroinflammation [4, 5] and the hippocampal metabolome (Fig. 3), system x_c_^−^ might be involved in shaping age-related changes in neuronal morphology or hippocampal function.

**Fig. 4:**
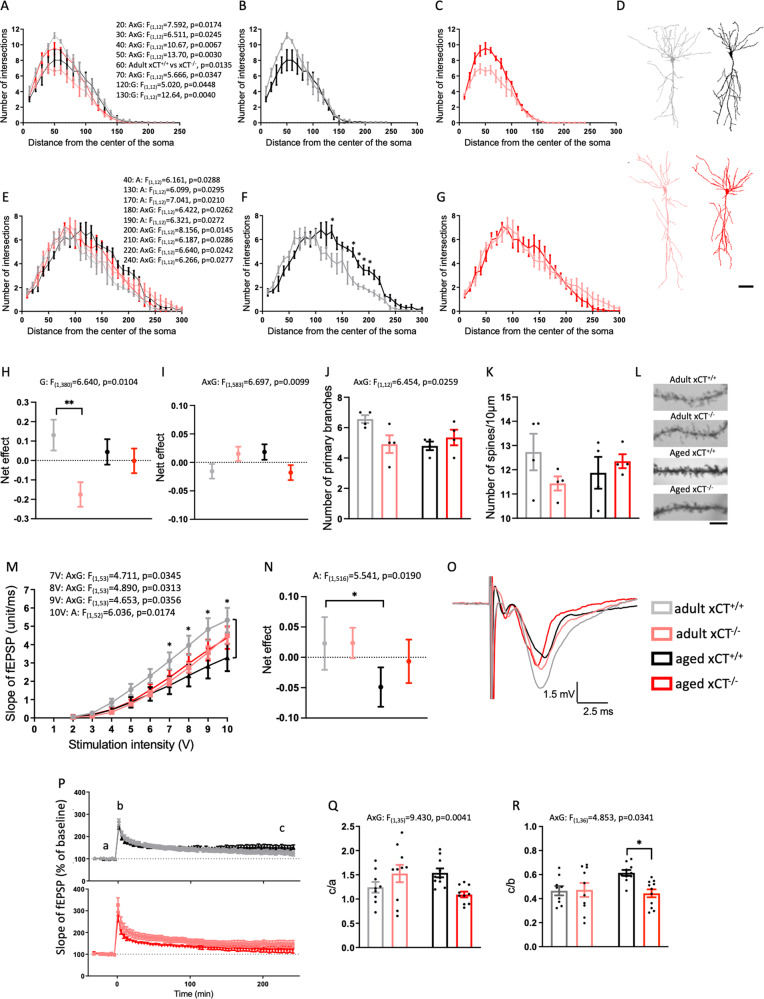
xCT deletion affects the age-related changes in dendritic arborization and protects against age-related impairment in hippocampal neurotransmission. Dendritic arborization of the basal (**A**–**C**, **H**) and apical tree (**E**–**G**, **I**) of CA1 pyramidal neurons was evaluated using a Sholl analysis (*n* = 4 mice/group; 17–18 neurons/group; representative tracings, **D**). The net effect represents the average of each group, corrected for the co-variate ‘distance from the center of the soma’ and relative to the average response of all groups (**H**, **I**). The number of primary branches was counted (**J**) and on each neuron spine density was calculated on 3–4 segments (total of 63–77 segments/group; **K**). Representative pictures of the dendritic spines (**L**). To generate input/output (I/O) curves, the mean of 2 electrodes/slice and 1–2 slices/animal was used (**M**; 13 slices from 7 adult xCT^+/+^ mice, 17 slices from 9 adult xCT^−/−^ mice, 11 slices from 6 aged xCT^+/+^ mice, 16 slices from 9 aged xCT^−/−^ mice). The net effect represents the average response of each group, corrected for the co-variate ‘stimulation intensity’ and relative to the average response of all groups (**N**). Representative tracings of fEPSP (stimulation of 8 V, **O**). LTP measurements in 9 slices from 7 adult xCT^+/+^ mice, 10 slices from 8 adult xCT^−/−^ mice, 10 slices from 6 aged xCT^+/+^ mice, 11 slices from 8 aged xCT^−/−^ mice (**P**–**R**). The ratio of the normalized fEPSP slope 4 h after the high-frequency stimulation over the slope before (**Q**) and immediately after (**R**) the stimulation, was used to quantify LTP. Data are presented as mean ± SEM. To calculate the net effect for the Scholl analysis and I/O curves, a longitudinal analysis was performed using a backward selected model and a correction for the respective co-variates, with an autoregressive model followed by a two-way ANOVA and a one-sided Sidak’s multiple comparisons test (**H**, **I**, **N**, see Supplementary Methods). All other data are analyzed using a two-way ANOVA or a Kruskal–Wallis test in case of non-normal distributed data (**A**–**C**, distance 60, 160, and 170) (see Supplementary Table 4): A aging effect, G genotype effect, AxG interaction effect; Sidak’s multiple comparisons; *p < 0.05; **p < 0.01. Scale bar: 50 μm (**D**), 5 μm (**L**).

We used Golgi-Cox staining to study the morphology and spine density of the CA1 pyramidal neurons. Loss of xCT reduced the complexity of the basal tree in adult mice (Supplementary Fig. 8A, B) and affected the age-related changes in dendritic arborization of both the basal and apical tree (Fig. 4A, E). Although not supported by significant post hoc comparisons, the number of intersections of the basal tree decreased with aging in xCT^+/+^ mice (Fig. 4B) while the opposite was observed in xCT^−/−^ mice (Fig. 4C), resulting in overlapping Sholl analysis profiles of adult xCT^+/+^ and aged xCT^−/−^ mice (Supplementary Fig. 8C). A longitudinal analysis that corrects for the co-variate ‘distance from the soma’ (see Supplementary Methods for details), shows an overall genotype effect for the basal tree (Fig. 4H). However, post hoc analysis only indicated a significant difference in adult mice (Fig. 4H). In the apical tree, we observed overall aging and interaction effects in the number of intersections at distances further away from the soma (Fig. 4E). Post hoc analysis revealed significant aging effects in xCT^+/+^ (Fig. 4F) but not in xCT^−/−^ mice (Fig. 4G). The longitudinal analysis of the entire apical tree (see Supplementary Methods for details) showed a significant interaction effect (Fig. 4I). While an interaction effect was detected in the number of primary branches (Fig. 4J), neither aging nor genotype affected the density of the spines on the apical tree (Fig. 4K, L).

We next performed slice electrophysiology to investigate functional changes at hippocampal CA3-CA1 connections, by stimulating Schaffer collaterals and recording the fEPSP from CA1 pyramidal neurons. We observed an interaction effect in the I/O curves that represent basal hippocampal neurotransmission, implying that system x_c_^−^ deficiency affects the age-related changes that were seen at higher stimulation levels (7–9 V), as well as an aging effect at 10 V (Fig. 4M). For each of the stimulation intensities, post hoc analysis revealed a significant age-related decrease in fEPSPs only in the xCT^+/+^ mice (Fig. 4M). A longitudinal analysis that corrects for the co-variate ‘stimulation intensity’ (see Supplementary Methods for details) did not allow to distinguish xCT^+/+^ from xCT^−/−^ mice and showed an overall age-related change in the output signal, that was again mainly driven by the xCT^+/+^ mice (Fig. 4N).

To study the ability of the Schaffer collaterals to generate LTP, we applied a high-frequency stimulation to the slices. We could induce and maintain LTP in slices of all groups (Fig. 4P). The age-related changes in LTP observed in xCT^+/+^ mice were absent in xCT^−/−^ mice (Fig. 4Q, R).

### xCT^−/−^ mice are protected against age-induced impairment in hippocampus-dependent memory

**Fig. 5:**
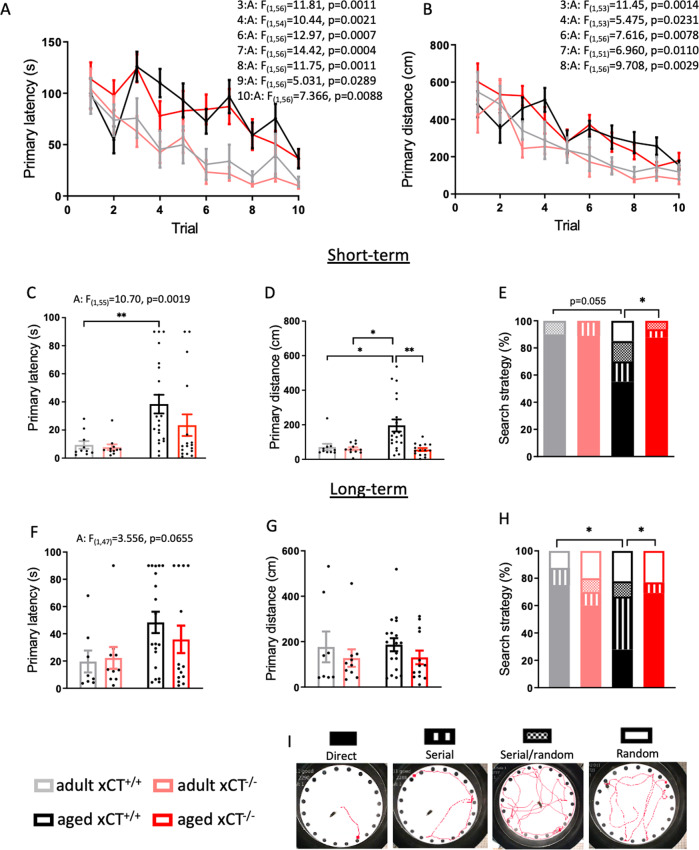
Absence of xCT protects against age-induced impairment of hippocampus-dependent memory. During the consecutive training sessions of the Barnes maze task, both primary latency (**A**) and distance (**B**) were studied as a parameter for learning (*n* = 11 adult mice/group; *n* = 17–21 aged mice/group). Short- (**C**, **D**) and long-term (**F**, **G**) memory was analyzed using the same parameters during a test trial respectively 24 h and 6 days after the last training session. The strategy that is used to locate the escape hole was evaluated during both test trials (**E**, **H**). A representative path for each search strategy is shown (**I**). Data on primary distance and latency are presented as mean ± SEM and analyzed using a two-way ANOVA followed by Sidak’s multiple comparisons test, or a Kruskal–Wallis test in case of non-normal distributed data (**D**) (see Supplementary Table 4): *p < 0.05; **p < 0.01. Significant main effects are presented in the figure: A aging effect. Data on the search strategy are presented as percentage of mice within each group that use the respective strategies; statistical analysis was performed using the Chi-square test focused on the direct strategy: *p < 0.05.

Finally, we evaluated whether the effects of system x_c_^−^ deficiency on the age-related changes at the level of the hippocampus -as described above- are translated into differences in memory function. We therefore compared the performance of adult and aged xCT^+/+^ and xCT^−/−^ mice in the Barnes maze task, to evaluate the effect of xCT deletion on age-related changes in hippocampus-dependent memory. Already after the first day of training, adult mice were more efficient in finding the escape hole compared to aged mice (Fig. 5A, B). The performance of all mice gradually improved and at day 5 (session 10) they all traveled the same distance to find the escape hole (Fig. 5B), indicating that also the aged mice learned the task. We next investigated short- and long-term memory of the mice, by evaluating their performance in a test trial at respectively 24 h and 6 days after the last training session. Aging increased the primary latency 24 h after the last training session, an effect that was mainly present in xCT^+/+^ mice (Fig. 5C). At this timepoint, aged xCT^+/+^ mice traveled a longer distance compared to adult mice and age-matched xCT^−/−^ mice, indicating a reduced performance of aged xCT^+/+^ mice compared to all other groups (Fig. 5D). The age-related effects on these parameters faded during the long-term test trial (Fig. 5F, G). During the test trials, we studied the search strategy that mice used to localize the target hole. Mice with intact spatial memory -which is hippocampus-dependent and particularly vulnerable to aging [45, 46]- use the direct search strategy (Fig. 5I). Contrary to wild-type mice, the majority of aged xCT^−/−^ mice continued to use the direct search strategy both at 24 h and 6 days after the last training session (Fig. 5E, H). These findings cannot be biased by differences in motor function or visual acuity, as we previously observed that the age-related decline in these functions was unaffected by xCT deletion [47]. Therefore, they unambiguously indicate improved hippocampus-dependent memory in mice that age in the absence of system x_c_^−^.

## Discussion

Genetic deletion of xCT results in life- and healthspan extension in mice, contrary to previous predictions [6] and despite a more prominent age-related oxidative shift in the plasma cystine/cysteine redox couple. The latter was not accompanied by changes in the GSSG/GSH ratio. This differential regulation of both disulfide/thiol redox couples has been described before and emphasizes that oxidative stress cannot be defined merely by the balance of each individual couple [48]. Although oxidative shifts in the plasma redox state have been correlated to pathological changes that are typically seen in aged organisms [49–51], age-related changes in clinical frailty index, sarcopenia or dynapenia remained unaffected by the loss of system x_c_^−^. Also age-related changes in general blood parameters were unaltered, except for fasting glucose levels that were slightly improved when mice age in the absence of system x_c_^−^. Altogether, these observations add to the multitude of findings that have been challenging the “free radical theory of aging” proposed by Harman [52] and support the more recent hypothesis of hormesis, stating that mild levels of oxidative stress are beneficial for life- and healthspan [53, 54], and might even counter some of the negative effects attributed to the reductive stress observed in the aged mice [42].

Redox perturbations and inflammation are hallmarks of aging and cognitive dysfunction [55] and a better balance between inflammaging and anti-inflammaging is suggested to be key in longevity control [13]. We here provide evidence for an involvement of system x_c_^−^ in the process of age-related priming of the innate immune system, a feature that is linked to increased susceptibility to different age-related disorders [56, 57] and a mechanism that could underly the increased lifespan in mice with a genetic deletion of xCT. While we were unable to associate the reduced priming in xCT^−/−^ mice to major changes in inflammatory markers in the hippocampus -a brain region particularly vulnerable to aging and important for memory function- a clear distinction in the metabolic profile of aged mice of both genotypes could be made. Different phospholipid species were normalized to adult levels in aged xCT^−/−^ mice. Phospholipids are important components of myelin and the cell membrane, but also participate in many cellular processes, including oxidative stress, inflammation and neurodegeneration [58, 59]. Several studies have shown aberrant phospholipid metabolism in aging, with decreases of different species and a negative impact on brain function and neuronal plasticity [59–62]. Decreased brain levels of plasmalogens, a subclass of phospholipids, have been correlated with functional decline in Alzheimer’s disease patients [63]. In contrast to wild-type mice, aged xCT^−/−^ mice did not show a decrease in plasmalogen levels.

Lack of xCT also prevented the age-induced increase in monoacylglycerol levels, including the endocannabinoid 2-arachidonoyl-glycerol, that was seen in aged xCT^+/+^ mice. This effect could be related to the reduced extracellular glutamate levels and consequently the decreased stimulation of metabotropic glutamate receptors mGluR1 and mGluR5, which are directly linked to the endocannabinoid system [64–66]. Elevated monoacylglycerols are seen in age-related neuropathologies [67, 68], and appear early in human Alzheimer’s disease pathology [68]. Moreover, disruption of the metabolism of 2-arachidonoyl-glycerol can affect the temporal and spatial control of retrograde signaling leading to exacerbation of synapse impairment in Alzheimer’s disease [69].

Advanced glycation end products (AGE) are formed via non-enzymatic chemical reactions between sugars and proteins or lipids. They are established markers of aging and known risk factors for the development of cognitive decline [70]. Similarly, carbamylation is a nonenzymatic degenerative protein modification known to accumulate in tissue over time [71], and reported to be associated with neuroinflammation and to contribute to the neuropathology of dementia [72]. xCT deletion beneficially affects the levels of markers for both AGE (N6-carboxymethyllysine) and carbamylation (homocitrulline). Finally, both acetylcholine -involved in learning and memory [73]- and DHEA-S-having anti-inflammatory and neurotrophic properties [74]- were increased in aged xCT^−/−^ mice, reiterating improved hippocampal function when mice age in the absence of xCT [73–75].

The above-described metabolic changes in the aged hippocampus, and the fact that both inflammation [76] and increased extracellular glutamate concentrations [77] can promote age-related changes in neuronal morphology and functionality, predict positive effects of system x_c_^−^ deficiency on the aging-associated dysregulated excitability in hippocampal pyramidal neurons [15]. Indeed, the conventional age-related impairments detected in the I/O curves generated in xCT^+/+^ mice, could not be seen in xCT^−/−^ mice. Also, LTP has been reported to be enhanced in aging when using a weak stimulus [78]; a phenomenon that has been linked to altered glutamate distribution in aged mice. Indeed, the NMDA receptor antagonist memantine that preferably blocks extrasynaptic receptors [79], was shown to decrease LTP induced by a weak stimulus in slices from aged but not adult mice [78]. It was therefore hypothesized that the increased LTP in aged mice results from an age-induced alteration in extrasynaptic glutamate regulation [21], leading to overactivation of -mainly extrasynaptic- NMDA receptors [78]. The latter would be attenuated in the absence of system x_c_^−^, which could explain the lower LTP in the aged xCT^−/−^ mice compared to aged xCT^+/+^ mice. Moreover, it has been shown that the increased amplitude of LTP in aged mice is accompanied by a loss of synapse specificity involving calcium channels and calcium release from the endoplasmic reticulum [33]. This loss of specificity of synaptic plasticity at the level of the aged hippocampus could result in memory impairment, as seen in aged xCT^+/+^, but not xCT^−/−^, mice.

Although inconsistent findings have been reported [80], a regression in the dendritic arbors of hippocampal CA1 neurons may underlie the first signs of decline in learning and memory performance associated with normal aging [81–83]. While adult xCT^−/−^ mice had a less complex basal tree compared to xCT^+/+^ mice, the complexity of the basal tree increased with aging in xCT^−/−^ mice. The age-related increase in arborization of the apical tree of xCT^+/+^ mice -once again an event which was not seen in xCT^−/−^ mice- was restricted to the mid-apical dendrites (*stratum radiatum*) where the CA1 neurons receive extensive glutamatergic input of the Shaffer collaterals, possibly representing a compensatory reaction induced by excitotoxic damage in the aged hippocampus, as described in ischemia [84]. While a more complex apical part of the CA1 pyramidal neurons was correlated with better reference memory in adult rats, in aged rats this correlation was inverted [85]. This is in line with our results showing age-induced deficits in hippocampus-dependent memory functions in xCT^+/+^ mice, while aged xCT^−/−^ mice retained their hippocampal memory. It should be noted that we previously were unable to observe differences in spatial reference memory between adult and aged xCT^−/−^ mice and xCT^+/+^ littermates in a Morris water maze task. However, the training protocol with short inter-trial intervals that was used in this study was likely biased towards spatial working memory performance which may have masked genotype differences in spatial reference memory. Indeed, we found that spatial working memory was significantly impaired in adult but not in aged xCT^−/−^ mice compared to their age-matched xCT^+/+^ controls. Moreover, given that adult and aged mice were studied and analyzed separately, we were unable to directly confirm a reduced age-related decline in memory performance in xCT^−/−^ mice compared to xCT^+/+^ littermates [7]. In the current study, we directly compared the performance of adult and aged xCT^+/+^ and xCT^−/−^ mice in the Barnes maze task, using a protocol with longer inter-trial intervals to avoid bias due to differences in spatial working memory, to specifically assess the effect of xCT deletion on age-related changes in hippocampus-dependent reference memory.

As described above, the reduced extracellular glutamate levels in the hippocampus of aged xCT^−/−^ mice [7] might mitigate the glutamatergic dysregulation in the aging brain and could -besides the observed metabolic changes- underlie the observed protection against age-related morphological and functional changes in hippocampal neurons, thereby preventing memory decline. Accordingly, riluzole-induced reduction in extracellular glutamate levels could prevent hippocampal cognitive decline in aged rats [77] and improved memory retrieval in aged growth hormone receptor knockout mice was correlated with decreased hippocampal glutamate levels [86]. D-galactose administration, a commonly accepted model for aging, has been associated with memory decline combined with increased hippocampal glutamate, while reducing glutamate levels in this model improved memory performance [87]. In addition, reduced AMPA receptor endocytosis, as reported in the hippocampus of xCT^−/−^ mice [3], was shown to protect against dysfunctional LTP as well as memory impairment [88]. Finally, reduced glutamate levels could also contribute to the increased lifespan observed in xCT^−/−^ mice, since longevity has been correlated with downregulation of genes involved in excitatory neurotransmission in humans and inhibition of excitation in glutamatergic neurons resulted in increased lifespan in *Caenorhabditis elegans* [89].

The current manuscript reports on the first elaborate study of the effects of system x_c_^−^ deficiency on the aging process of male mice, while gender differences have been reported both in the age-related changes in the immune response, in age-related frailty and cognitive impairment (for review [90]), and in the reaction of the brain to genetic xCT deletion [91, 92]. Our conclusions also rely on observations in a single transgenic mouse model with a genetic deletion of xCT [6]. Another mouse strain that has been used to study the effects of xCT deficiency, is the subtle gray (*sut/sut*) mutant. This strain carries a large spontaneous mutation that includes -but also extends beyond- exon 12 of the xCT-encoding gene [44, 93]. Although some findings on hippocampal and cognitive function that have been reported in the *sut* mice [94–96] are conflicting with those in the xCT^−/−^ mice, these discrepancies seem absent when comparing the *sut* mice with littermate controls [91, 92]. Still, it would be of interest to confirm the major findings of the current study both in female mice and by using different strategies to target system x_c_^−^. Finally, for reasons that have been explained throughout the paper, we focused our analyses on the hippocampus. The effect of xCT deletion on age-related changes in other brain regions that are important for memory function, such as the prefrontal cortex, therefore awaits further investigation.

To conclude, we report for the first time that male mice that age in the absence of system x_c_^−^ have an extended lifespan, have reduced priming of the peripheral immune system and are protected against age-related decline in hippocampal function and memory, a feature of aging with a devastating impact on the elder. Moreover, we anticipate that reduced priming of the peripheral innate immune system in aged xCT^−/−^ mice, as well as the improved metabolic state of the aged xCT^−/−^ hippocampus, will also be beneficial for other age-related impairments that have not been addressed in the current study.

## Supplementary information

Supplemental material

## Data Availability

Supplementary Information is available at MP’s website. The metabolomics dataset is accessible via Mendeley Data (10.17632/hb947bsgh6.1).
