# Supplementary material for: Lifespan extension with preservation of hippocampal function in aged system xc−-deficient male mice
Source: Mol Psychiatry. 2022 Feb 18;27(4):2355–68. doi: 10.1038/s41380-022-01470-5 (PMC9126817; doi:10.1038/s41380-022-01470-5)
Supplement: Supplementary file 1 — Supplemental material [file 41380_2022_1470_MOESM1_ESM.docx]

Supplementary data for

**Lifespan extension with preservation of hippocampal function in aged system x_c_^-^ - deficient male mice**

Lise Verbruggen^†^, Gamze Ates^†^, Olaya Lara, Jolien De Munck, Agnès Villers, Laura De Pauw, Sigrid Ottestad-Hansen, Sho Kobayashi, Pauline Beckers, Pauline Janssen, Hideyo Sato, Yun Zhou, Emmanuel Hermans, Rose Njemini, Lutgarde Arckens, Niels C. Danbolt, Dimitri De Bundel, Joeri L. Aerts, Kurt Barbé, Benoit Guillaume, Laurence Ris, Eduard Bentea^†^, Ann Massie^†^*

^†^Equally contributing first and senior authors

*Corresponding author. Email: ann.massie@vub.be

**This file includes:**

Supplementary Methods

Supplementary Results

Supplementary Figures 1 to 8

Supplementary Tables 1 to 4

References

Supplementary Methods

Clinical chemistry

The ion concentration (K^+^, Cl^-^, Na^+^) was determined by the difference in electrical potential (electromotive force) between a solution of known ion concentration and the test sample, and the value of the electromotive force was obtained by the Nernst equation for a given ion. Total albumin was quantified by an immuno-turbidimetric method in which anti-albumin antibodies will complex with albumin and the total protein concentration was obtained using the colorimetric Biuret method. Urea, aspartate aminotransferase (AST), alanine aminotransferase (ALT) and lactate dehydrogenase (LDH) levels were measured based on photometrically analysis of NADH levels, since all these enzymes will induce a final oxidation of NADH into NAD^+^. The determination of total bilirubin, alkaline phosphatase (ALP) and pancreatic α-amylase concentration is based on a colorimetric method which measures released/formed azobilirubin for total bilirubin or p-nitrophenol for ALP and α-amylase. The creatinine concentration is determined by the Jaffé method with "rate-blanking" to reduce interference from bilirubin and a correction factor of -0.3mg/dL. Finally, total cholesterol, high density lipoprotein-cholesterol (HDL) and triglycerides concentrations are measured by an enzymatic colorimetric method that results in the production of hydrogen peroxide which reacts with 4-aminophenazone to form a red dye. The concentration of non-HDL is obtained by subtracting HDL from total cholesterol and the concentration of low-density lipoprotein (LDL)-cholesterol is obtained by the Friedewald formula. Plasma lactate and pyruvate levels were measured using the colorimetric detection-based kits MAK329 and MAK332 (Sigma-Aldrich), respectively, according to manufacturer’s instructions.

Flow cytometry

Spleens were harvested and gently homogenized through a cell strainer (40µm nylon, CORNING Life Sciences). Erythrocytes were removed using red blood cell lysis buffer (150mM NH_4_Cl, 10mM KHCO_3_ and 0.13mM EDTA; pH 7.2) and 1.5*10^6^ cells were stained with fixable viability stain-780 (Sigma-Aldrich*,* 1/2000 in 2mM EDTA in 10mM PBS) for 20min in the dark at room temperature. Next, cells were stained with antibodies against cell surface markers for T cells, NK cells and various myeloid cell populations (Supplementary Table 2A) for 20min in the dark at 4°C. Cells were acquired using a BD LSR Fortessa cytometer (BD Biosciences). All read-outs had control samples of matching cells that were unstained to rule out auto-fluorescence and Fluorescence Minus One controls were used for set-up. Data were analysed using Flowlogic software (Miltenyi Biotech); the gating strategy is shown in Supplementary Figure 5.

Immunofluorescence

Mice were deeply anesthetized using pentobarbital (Dolethal^®^, 200mg/kg i.p.) and transcardially perfused with 4% formaldehyde (10ml/min for 10min). Brains were immersed in the same fixative for 2h and transferred to a 10% sucrose solution (in 100mM PBS) for 4h at 4°C, followed by a 20% sucrose solution overnight. The next day, brains were transferred to a 30% sucrose solution containing 0.02% sodium azide, frozen on dry ice and sliced into coronal sections of 30-40µm. Immunofluorescent labeling for xCT (RRID:AB_2714118) and double labeling with xCT and GLAST (EAAT1, RRID:AB_2714072) as well as xCT and Iba-1 (RRID:AB_2619949) were performed as described [1], using antibodies as detailed in Supplementary Table 2B. For the xCT staining, sections of xCT^-/-^ mice were simultaneously processed to rule out non-specific labeling. Labeled sections were examined with a Zeiss Axioplan 2 microscope equipped with a Zeiss LSM 510 meta confocal scanner head (Carl Zeiss Microscopy GmBH). The original images were assembled using the Adobe InDesign CS6 software.

Western blotting

Mice were sacrificed by cervical dislocation and hippocampus was snap frozen. Proteins were extracted and hippocampal xCT expression levels were quantified relative to a pooled sample using semi-quantitative Western blotting, as described before [2, 3], and using the antibodies as specified in Supplementary Table 2B. Hippocampal extracts of xCT^-/-^ mice were blotted on the same membrane as a negative control.

[^3^H]-L-glutamate uptake in hippocampal synaptosomes

Hippocampi were mechanically homogenized in ice-cold sucrose (320mM) and centrifuged at 1000g for 10min at 4°C. The supernatants were collected and centrifuged again for 30min at 17500g. The final pellet containing the synaptosomes was suspended in ice-cold Na^+^-free buffer (140 mM N-methyl-D-glucamine, 5.4mM KCl, 2.5mM CaCl_2_, 1mM MgCl_2_, 0.4mM KH_2_PO_4_, 10mM HEPES and 5mM D-glucose; pH 7.4) and the total protein concentration determined with the Bio-Rad protein assay (Bio-Rad Laboratories). [^3^H]-L-glutamate (specific activity of 48.6Ci/mmol, PerkinElmer) was used as substrate at a tracing concentration of 20nM to evaluate the reversed transport carried out by system x_c_^-^. In a total volume of 500μL Na^+^-free buffer, 10μg of protein (synaptosome preparation) was incubated with [^3^H]-L-glutamate at 37°C, in the presence or absence of homocysteic acid (HCA, 1µM). After 20min incubation at 37°C, the suspension was filtered through a GF/B glass fibre filter adapted to a 96-well plate (UniFilter GF/B, PerkinElmer), and washed three times with ice-cold Na^+^-free buffer. After drying overnight at room temperature, Microscint 20 (PerkinElmer) was added to each well of the filter plate. Plates were shaken for 2h and counted with a Topcount^®^ NXT Microplate scintillation and luminescence counter (PerkinElmer). Results are expressed as cpm per μg of protein. System x_c_^-^-specific [^3^H]-L-glutamate uptake was calculated by subtracting the uptake in the presence of the inhibitor HCA from the total uptake, in the absence of Na^+^. Specificity of the uptake was further confirmed by the absence of activity in xCT^-/-^ samples.

Statistical analysis

The statistical model used can be positioned in the general linear model framework in particular “longitudinal design”. A longitudinal study is a cross-sectional design where a variable of interest is measured as a function of time/space. Different than in repeated measures, a longitudinal design applies an explicit equidistant grid on time/space. For this particular application we illustrate the statistical model using the data of the Sholl analysis of the basal tree (Fig. 4A, main text). Consider $y_{tik}$ the number of intersections for mouse $k$ in group $i$ which is either adult xCT^+/+^, adult xCT^-/-^, aged xCT^+/+^ or aged xCT^-/-^ for a specific distance $D_{t}$ from the centre of the soma, where $t$ is the index denoting the specific distance level. A classical model would imply the following main effects (ANCOVA) model:

(Eq 1) $y_{tik}=\mu+\theta_{i} X_{i}+\beta D_{t}+\epsilon_{tik}$

This model would not correct for the correlation of the data within the same mouse as a function of the distance from the centre of the soma. Indeed, the noise term $\epsilon_{tik}$ is correlated for different values of $t$ resulting in a bias. In this analysis, the correlation structure was analysed which is shown to exhibit an autoregressive structure. As a result, the model was enhanced by a second-order autoregression leading to:

(Eq 2) $y_{tik}=\mu+\theta_{i} X_{i}+\beta D_{t}+\alpha_{1}y_{t-1,i,k}+\alpha_{2} y_{t-2,i;k}+\eta_{tik}$

where the residuals $\eta_{tik}$ were tested to guarantee that these follow a Gaussian white distribution. This model can be further enhanced by inclusion of possible interaction effects leading to a full-factorial dynamic longitudinal model. In order to avoid overfitting, a backward elimination step was used to maximize the adjusted-R^2^ statistic; as illustrated in the table below (the terms which are eliminated by the backward elimination are not tested and marked by ***):

| ***Tests of Between-Subjects Effects:*** **Dependent Variable is the number of intersections** | | | | | |
| --- | --- | --- | --- | --- | --- |
| Source | Type III Sum of Squares | df | Mean Square | F | Sig. |
| Corrected Model | 4526,577a | 21 | 215,551 | 526,151 | 0,000 |
| Intercept | 205,211 | 1 | 205,211 | 500,911 | 0,000 |
| $X_{i}$ | 0,401 | 3 | 0,134 | 0,326 | 0,806 |
| $D_{t}$ | 172,886 | 1 | 172,886 | 422,008 | 0,000 |
| $y_{t-1,i,k}$ | 107,841 | 1 | 107,841 | 263,235 | 0,000 |
| $y_{t-2,i,k}$ | 4,417 | 1 | 4,417 | 10,781 | 0,001 |
| ${X_{i}\times y}_{t-1,i,k}$ | 1,492 | 1 | 1,492 | 3,642 | 0,057 |
| ${X_{i}\times y}_{t-2,i,k}$ | 6,753 | 3 | 2,251 | 5,495 | 0,001 |
| ${D_{t}\times y}_{t-1,i,k}$ | 1,938 | 3 | 0,646 | 1,577 | 0,195 |
| ${D_{t}\times y}_{t-2,i,k}$ | *** | 1 | *** | *** | p>0,05 |
| $X_{i}\times D_{t}$ | 25,200 | 1 | 25,200 | 61,512 | 0,000 |
| $y_{t-1,i,k}\times y_{t-2,i,k}$ | *** | 1 | *** | *** | p>0,05 |
| $D_{t}\times y_{t-1,i,k}\times y_{t-2,i,k}$ | *** | 1 | *** | *** | p>0,05 |
| $X_{i}\times D_{t}\times y_{t-1,i,k}$ | *** | 3 | *** | *** | p>0,05 |
| $X_{i}\times D_{t}\times y_{t-2,i,k}$ | 1,377 | 3 | 0,459 | 1,121 | 0,341 |
| $X_{i}\times y_{t-1,i,k}\times y_{t-2,i,k}$ | 9,492 | 4 | 2,373 | 5,793 | 0,000 |
| $X_{i}\times D_{t}\times y_{t-1,i,k}\times y_{t-2,i,k}$ | 148,303 | 362 | 0,410 |  |  |
|  | 8616,966 | 384 |  |  |  |
| Error | 4674,880 | 383 |  |  |  |
| Total | *** | 1 | *** | *** | p>0,05 |
| Corrected Total | *** | 1 | *** | *** | p>0,05 |
| a. R Squared = ,968 (Adjusted R Squared = ,966) |  |  |  |  |  |

****: Terms which are eliminated by the backward elimination*

The model diagnostics are verified such that the model residuals satisfy the assumptions of (i) linearity, (ii) homoscedasticity and (iii) normality. This leads respectively to the following diagnostic plots:


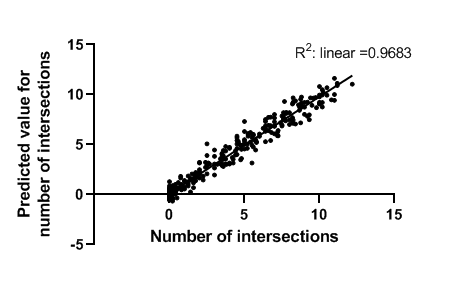


The plot checks that the model is unbiased such that the model predictions correspond to the empirical measurements assessing the model correspondence.


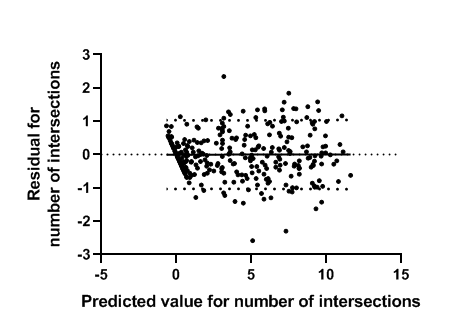


The plot checks the homoscedasticity of the model residuals which is consequently tested by Levene’s test showing that the homoscedasticity assumption is not significantly violated:

| Levene's Test of Equality of Error Variances | | | | | |
| --- | --- | --- | --- | --- | --- |
|  | | Levene Statistic | df1 | df2 | Sig. |
| Residual for Neuron | Based on Mean | 2,363 | 3 | 380 | 0,071 |
|  | Based on Median | 2,243 | 3 | 380 | 0,083 |
|  | Based on Median and with adjusted df | 2,243 | 3 | 350,381 | 0,083 |
|  | Based on trimmed mean | 2,343 | 3 | 380 | 0,073 |

Finally, the distribution of the model residuals is verified to exhibit a normal distribution:


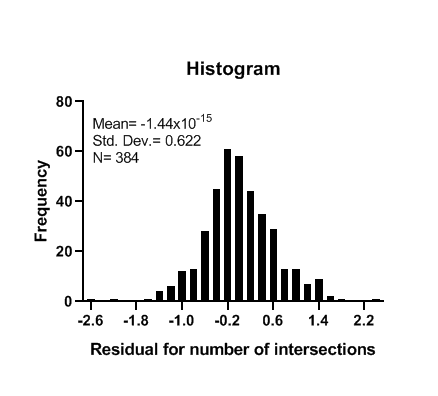


In a post-hoc step, the effect of type (adult xCT^+/+^, adult xCT^-/-^, aged xCT^+/+^ or aged xCT^-/-^) is analysed by studying the residuals of the model with regard to the terms which are unrelated to type and considered a confounder (marked in blue in the statistical table). These residuals are analysed through a two-way ANOVA as a function of type followed by Sidak's multiple comparisons test.

In case of the Sholl analysis, this longitudinal study corrects for the co-variate ‘distance from the soma’ and gives information on the basal or apical tree as an entity, thereby losing detailed information on the structure of the dendritic tree. Similarly, in case of the slice electrophysiology data, this longitudinal analysis corrects for the co-variate ‘stimulation intensity’ and gives information on the I/O curve as an entity, thereby losing detailed information on each stimulation intensity.

Supplementary Results and Figures

Criteria to quantify hippocampal gliosis

Iba-1^+^ microglia and GFAP^+^ astrocytes have been quantified in the dentate gyrus (Supplementary Figure 1 and Fig. 2 of the main text). Besides area and diameter, the convex closure was measured for Iba-1^+^ microglia (Supplementary Figure 1B). GFAP^+^ astrocytes were differentiated from GFAP^+^ progenitor cells based on location and morphology. While GFAP^+^ astrocytes are located in the hilus of the dentate gyrus and have processes in all directions (multipolar), GFAP^+^ progenitor cells are located in the subgranular zone and are predominantly bi- or unipolar, having one process that enters the molecular layer of the dentate gyrus [4] (Supplementary Figure 1C-E).

Supplementary Figure 1: Representative pictures of Iba-1 and GFAP immuno-histochemistry. The number and morphology of Iba-1^+^ cells in the dentate gyrus were studied (A, region of interest is indicated in blue). Besides the convex closure (B, black line), the area and diameter of the cell body were analyzed (B, red line). GFAP immunohistochemistry was performed to visualize astrocytes in the dentate gyrus (C, region of interest is indicated in blue). Astrocytes (C, black arrows; representative picture in D) were separated from GFAP^+^ progenitor cells (C, red arrow; representative picture in E) based on morphology and location [4]. The number of GFAP^+^ astrocytes was quantified, and their morphology evaluated by measuring area and diameter of the cell body (D, black line). Scale bar: 100μm in A, C; 20μm in B, D, E.

xCT deletion has no effect on the general health parameters

Despite an oxidative shift in the plasma cystine/cysteine couple (main text Fig. 1C-E) and an age-related decrease of plasma GSSG and GSH levels, no changes were detected in the plasma GSSG/GSH ratio (Supplementary Figure 2A-C). Both plasma lactate (Supplementary Figure 2D) and pyruvate (Supplementary Figure 2E) levels decreased with aging, while the lactate/pyruvate ratio was significantly increased in aged mice of both genotypes (Supplementary Figure 2F). Furthermore, clinical frailty at an age where the mice approached the end of their lifespan was similar in the xCT^+/+^ and xCT^-/-^ mice, notwithstanding an age difference (22-24 months for xCT^+/+^ mice and 24-28 months for xCT^-/-^ mice, Supplementary Figure 2G). Absence of xCT did not have a major impact on the body weight, the rectal temperature or organ and muscle mass (Supplementary Figure 2H-N).


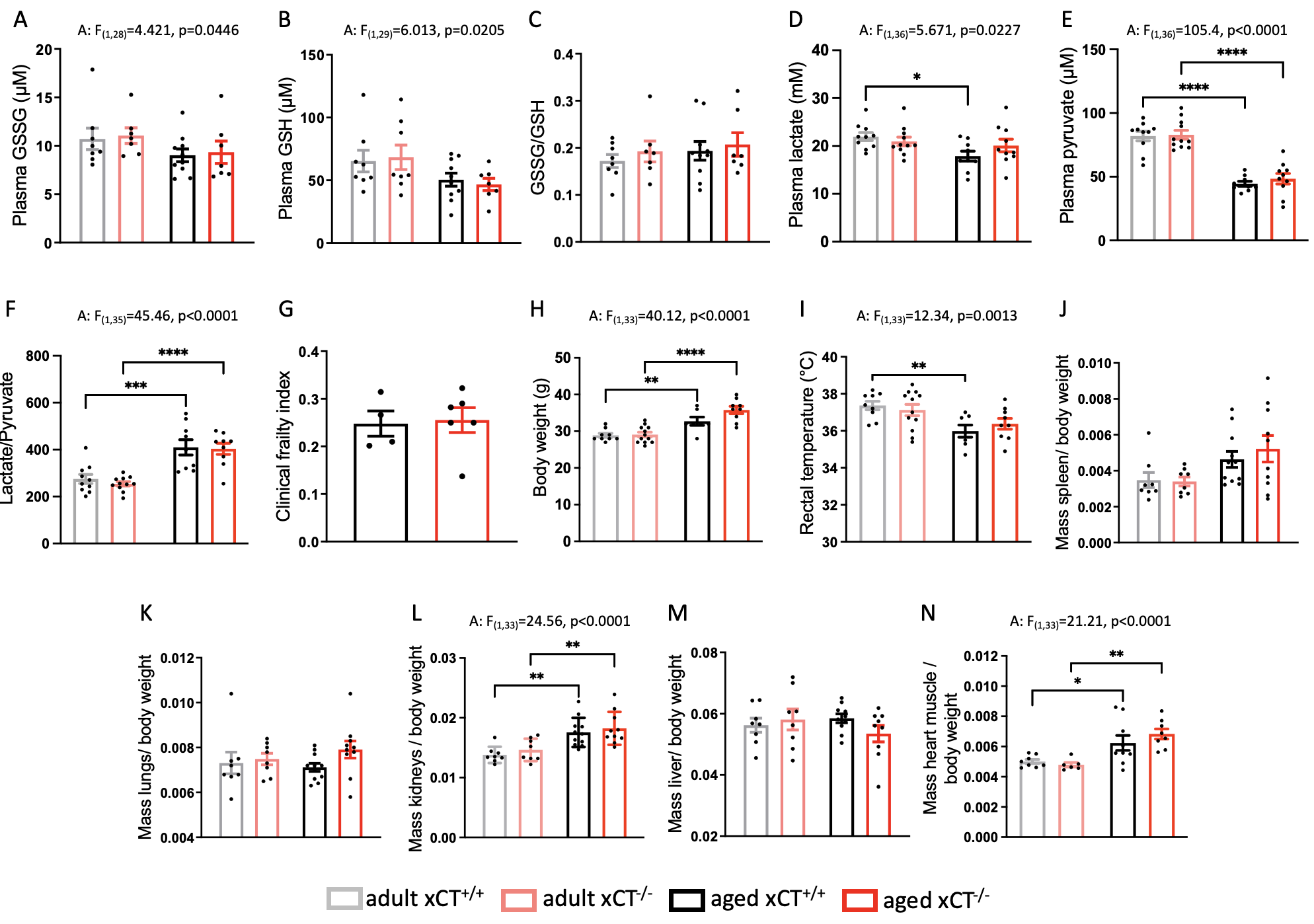


Supplementary Figure 2. Absence of xCT does not influence general health parameters. Plasma of adult and aged xCT^+/+^ and xCT^-/-^ mice was used to measure concentrations of glutathione disulfide (GSSG, A), glutathione (GSH, B), lactate (D) and pyruvate (E) and these values were used to calculate the GSSG/GSH (C) and lactate/pyruvate ratio (F), respectively (n=7-10 mice/group). The clinical frailty test was performed at 22-24 months for xCT^+/+^ mice and 24-28 months for xCT^-/-^ mice (G, n=4-6 mice/group). To further evaluate the general health status, body weight (H), body temperature (I) and organ weights (J-N) were studied (n=8-12 mice/group for adult mice; n=6-11 mice/group for aged mice). Data are presented as mean ± SEM and statistical analysis was performed using a two-way ANOVA followed by Sidak's multiple comparisons test, or a Kruskal-Wallis test in case of non-normal distributed data (J) (see Supplementary Table 4): ^*^p<0.05, ^**^p<0.01; ^***^p<0.001; ^****^p<0.0001. Significant main effects are presented in the figure: A: aging effect.

Most of the plasma parameters are affected by aging, but not genotype

Blood chemistry analysis indicated aging effects on plasma ion concentrations (Supplementary Figure 3A-C) and protein levels (Supplementary Figure 3D-E). Levels of LDH -a marker for tissue damage- and AST -a marker for lysis of liver, heart and skeletal muscle tissue- were unaltered by age and genotype (Supplementary Figure 3F-G). Aging affected markers for liver function (Supplementary Figure 3H-I). Moreover, an overall decrease in ALT values -a parameter for liver damage- was seen in the absence of system x_c_^-^ (Supplementary Figure 3I). Plasma concentrations of total bilirubin were increased in adult xCT^-/-^ mice compared to age-matched xCT^+/+^ mice and aged xCT^-/-^ mice, with the notion that two xCT^-/-^ mice showing high bilirubin levels (Supplementary Figure 3J) also showed high creatinine levels (Supplementary Figure 3K). High bilirubin levels were suggested to represent a protective mechanism in patients with high creatinine levels but normal renal function [5], as bilirubin has both anti-inflammatory and antioxidant properties [6]. An overall aging effect that was mostly driven by xCT^+/+^ mice was observed for urea and amylase (Supplementary Figure 3L-M). A significant age-related increase was seen in non-HDL cholesterol levels (Supplementary Figure 3R) while neither HDL-cholesterol, LDL-cholesterol nor triglycerides were influenced by aging or genotype (Supplementary Figure 3O-Q).


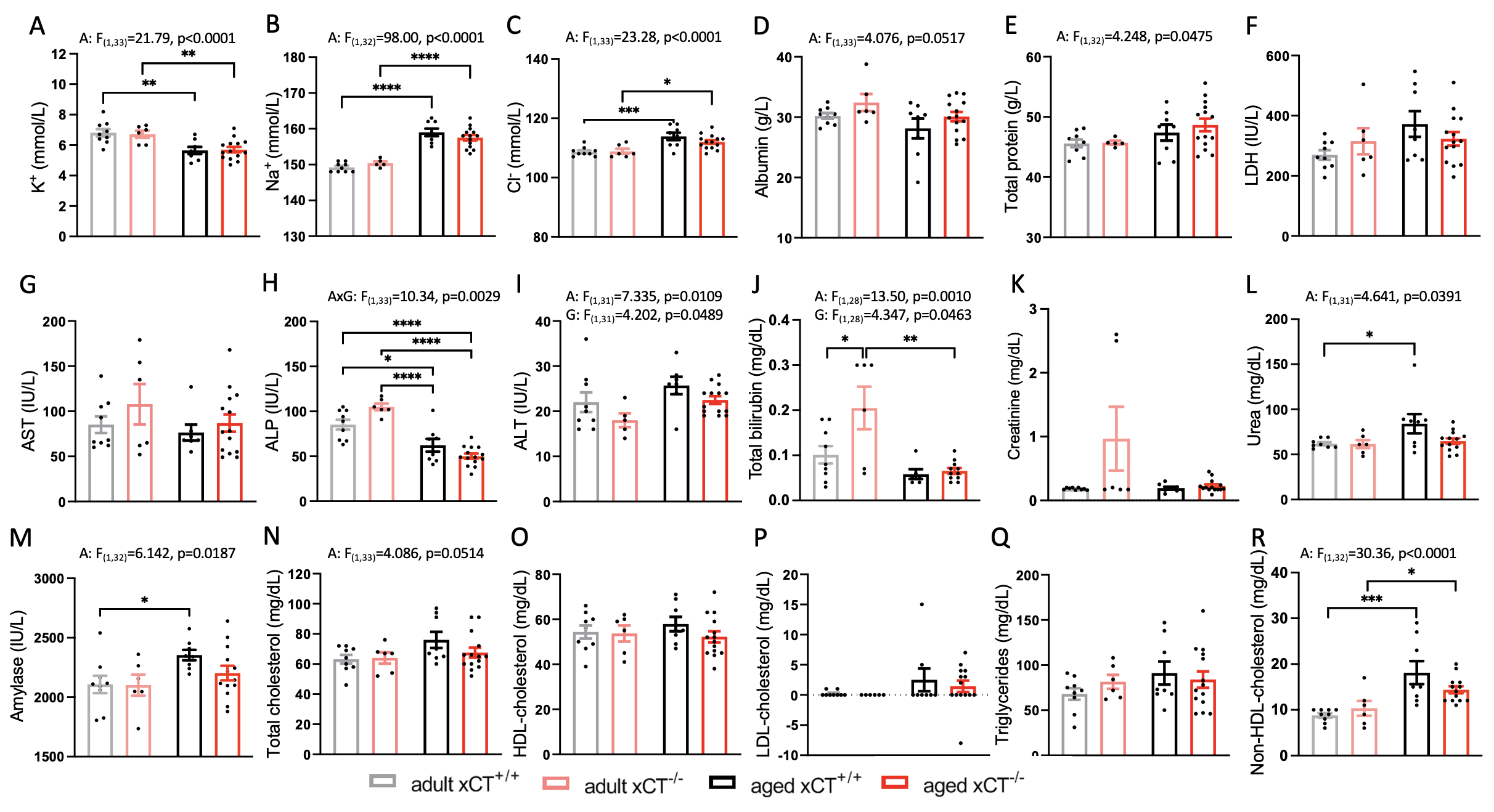


**Supplementary Figure** **3: Most of the plasma parameters are affected by aging, but not genotype***.* The plasma concentrations of K^+^ (**A**), Na^+^ (**B**) and Cl^-^ (**C**) reflect the ion homeostasis; albumin (**D**) and total protein levels (**E**) the protein content. Lactate dehydrogenase (LDH, **F**) and aspartate aminotransferase (AST, **G**) concentrations are a measure for tissue damage. Liver damage was analyzed by measuring alkaline phosphatase (ALP, **H**), alanine aminotransferase (ALT, **I**) and total bilirubin (**J**) levels. Levels of creatinine (**K**), urea (**L**) and amylase (**M**) were quantified. Lipid metabolism was studied using the plasma concentrations of total cholesterol (**N**), HDL-cholesterol (**O**), LDL-cholesterol (**P**), triglycerides (**Q**) as well as non-HDL-cholesterol (**R**). For all analyses, 6-9 adult and 8-14 aged mice of each genotype were used. Data are presented as mean ± SEM and analyzed using a two-way ANOVA followed by Sidak's multiple comparisons test, or a Kruskal-Wallis test in case of non-normal distributed data (**K,** **P**) (see Supplementary Table 4): ^*^p<0.05, ^**^p<0.01, ^***^p<0.001, ^****^p<0.0001. Significant main effects are presented in the figure: A: aging effect, G: genotype effect, AxG: interaction effect.

Aging, but not absence of xCT, changes the proportion of immune cells in the spleen

Absence of system x_c_^-^ did not alter the splenic population of T cells and innate immune cells. Also, the typical age-induced changes in these populations remained unaffected by xCT deletion (Supplementary Figure 4, 5). Aging decreased the percentage of viable CD3^+^ T cells (Supplementary Figure 4A), without affecting the relative proportion of CD4^+^ or CD8^+^ T cells in this fraction (Supplementary Figure 4B-C). Within both subpopulations, we observed an age-induced shift from naive to memory T cells, a typical feature of T-cell senescence [7] (Supplementary Figure 4D-I). No differences were seen in the effector CD8^+^ T-cell population (Supplementary Figure 4J), contrary to the proportion of CD4^+^ effector T cells for which we observed an age-induced increase that was mainly driven by the xCT^+/+^ mice (Supplementary Figure 4K). The percentage of non-cytotoxic NK cells within the viable CD3^-^ population decreased with aging, whereas the proportion of cytotoxic NK cells remained unaltered (Supplementary Figure 4L-M). As expected, due to increased extramedullary hematopoiesis during inflammation [8], the proportion of different myeloid cell types increased with aging (Supplementary Figure 4N-Q).


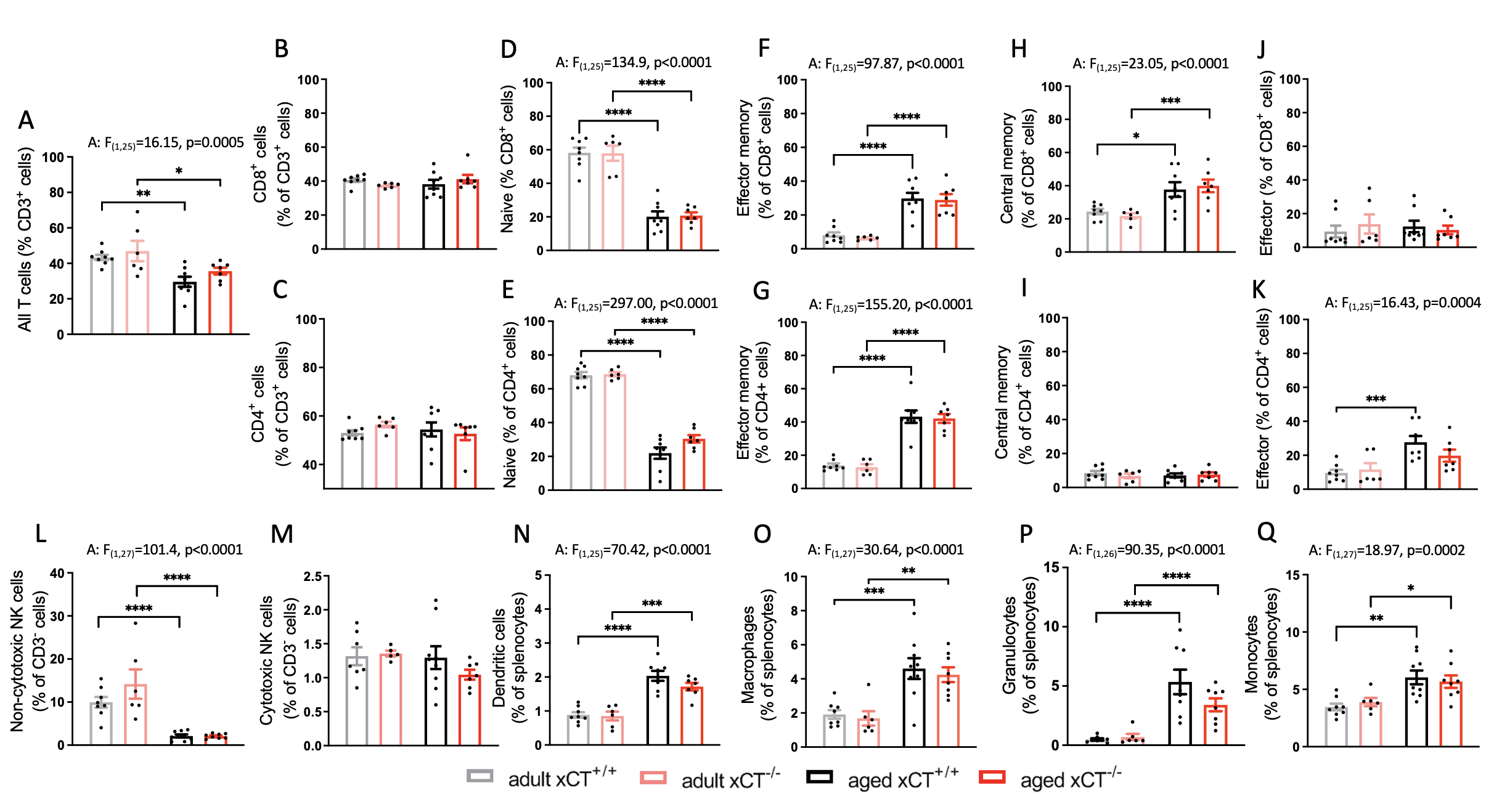


**Supplementary Figure** **4: Aging, but not absence of xCT, changes the proportion of immune cells in the spleen.** From the total living single-cell splenocyte population of adult and aged xCT^+/+^ and xCT^-/-^ mice (n=6-9 mice/group), we plotted the percentage of CD3^+^ cells (T cells, **A**). From this population, the fraction of CD8^+^ (**B**) and CD4^+^ T cells (**C**) was extracted, and within these subpopulations we studied the proportion of naive (**D, E**), effector memory (**F, G**), central memory (**H, I**) and effector T cells (**J, K**). From the viable CD3^-^ fraction, we plotted the percentage of non-cytotoxic (**L**) and cytotoxic NK cells (**M**). Finally, from the total single-cell population of splenocytes, we selected the dendritic cells (**N**), macrophages (**O**), granulocytes (**P**) and monocytes (**Q**). The markers used as well as the gating strategy for the different subpopulations are shown in Supplementary Figure 5 and Supplementary Table 2A. Data are presented as mean ± SEM and analyzed using a two-way ANOVA followed by Sidak's multiple comparisons test: ^*^p<0.05, ^**^p<0.01, ^***^p<0.001, ^****^p<0.0001, or a Kruskal-Wallis test in case of non-normal distributed data (**C**) (see Supplementary Table 4). Significant main effects are presented in the figure: A: aging effect.


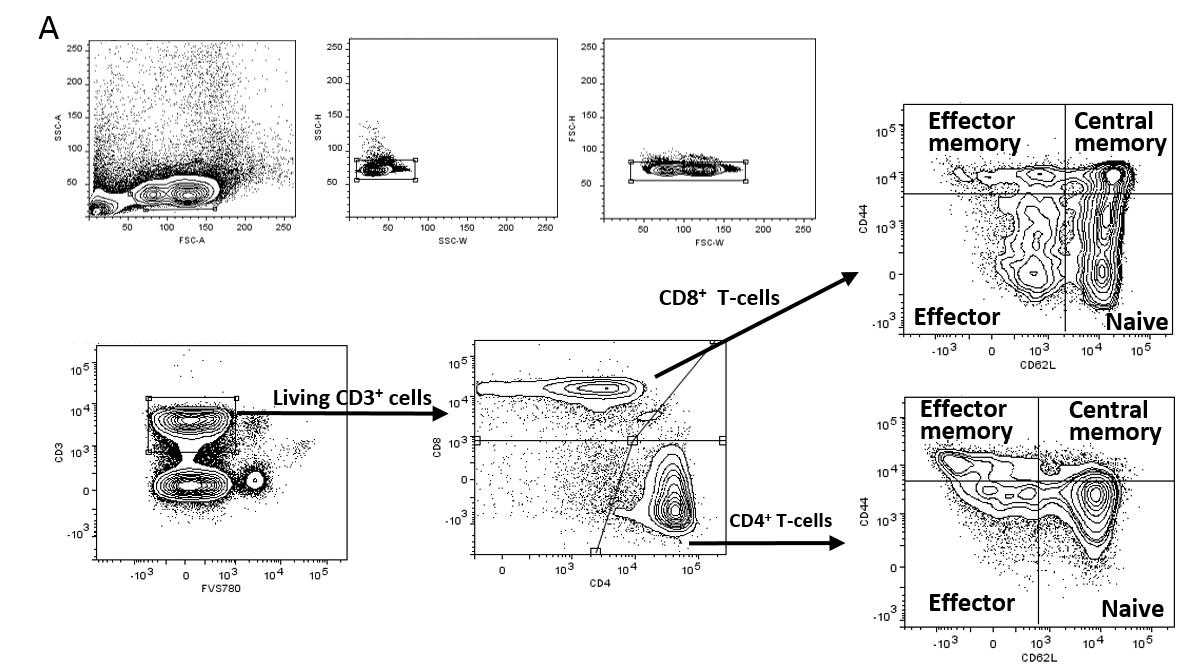

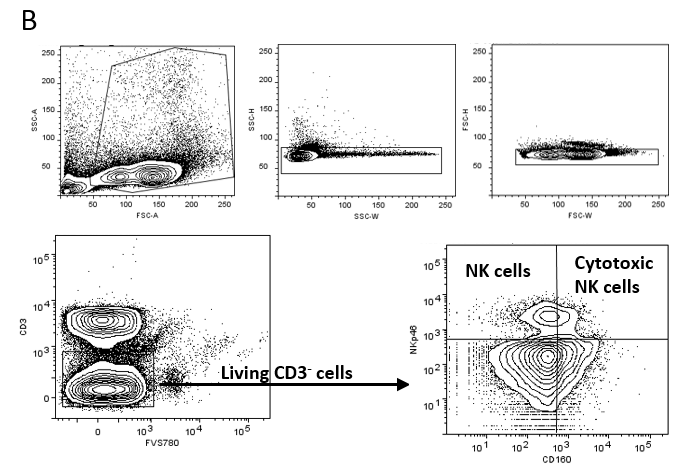


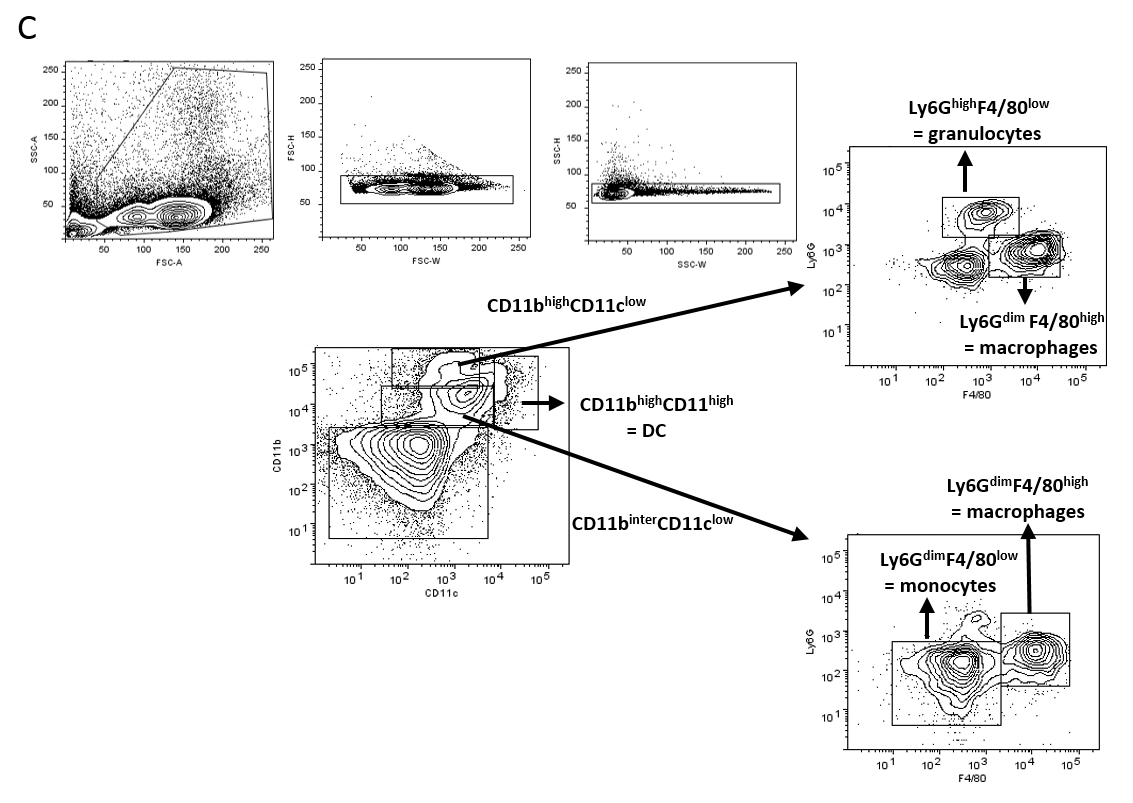


Supplementary Figure 5. Gating strategy for flow cytometry analysis of the immune cell populations in the spleen. The percentage of CD3^+^ cells (T cells) was extracted from the total living single-cell population of splenocytes. Within this population, the fraction of CD8^+^ and CD4^+^ T cells was selected and within these subpopulations the proportion of naive, effector memory, central memory and effector T cells were extracted using CD44 and CD62L cell surface markers (A). Next, from the viable CD3^-^ fraction, we selected the percentage of non-cytotoxic natural killer cells (NK cells) and cytotoxic NK cells using NKp46 and CD160 cell surface markers (B). Finally, from the total single-cell population of splenocytes, we selected the dendritic cells (DC) and other myeloid cells using CD11b and CD11c cell surface markers and within the other myeloid cells we selected the macrophages, granulocytes and monocytes using Ly6G and F4/80 cell surface markers (C).

Aging does not affect xCT distribution or protein expression in the mouse hippocampus

Cellular distribution in the hippocampus -a brain region particularly affected by aging- was analyzed using immunofluorescence. In all studied subregions, the aged hippocampus showed the same patchy xCT labeling pattern as reported for the adult mouse brain [1] and these xCT-positive patches were co-localized with an astrocytic (GLAST/EAAT1; Supplementary Figure 6E), but not with a microglial marker (Iba-1; Supplementary Figure 6F). Next, hippocampal xCT protein expression was measured using semi-quantitative Western blotting and shown to be unaltered with aging (Supplementary Figure 6G). Finally, system x_c_^-^ activity was quantified by measuring the reverse uptake of [^3^H]-L-glutamate in Na^+^-free medium to shut down the activity of the Na^+^-dependent excitatory amino acid transporters (EAATs), and in the presence of HCA, an inhibitor of system x_c_^-^. According to our observations on xCT expression, equal system x_c_^-^ activity was seen in synaptosomes of adult and aged hippocampus (Supplementary Figure 6H). Absence of signal in xCT^-/-^ samples confirmed specificity of all analyses described above.


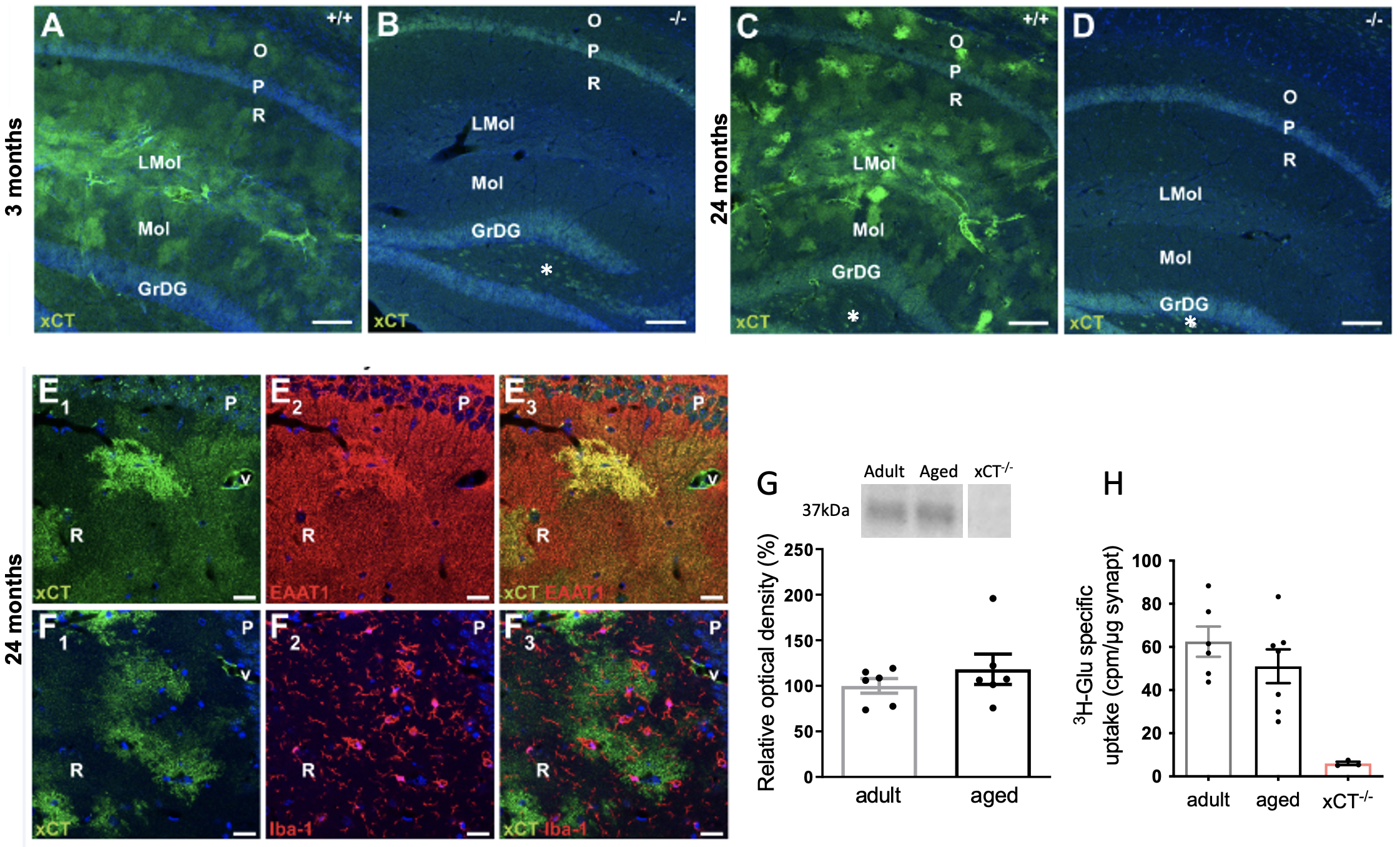


**Supplementary Figure** **6: Aging does not affect xCT distribution or protein expression in the mouse hippocampus.** xCT distribution was studied in adult (**A**) and aged mice (**C**, **E-F**) using immunofluorescence. xCT^-/-^ mice were used as negative control (**B, D**). Some unspecific nuclei staining that is also present in the xCT^-/-^ sections is indicated with an asterisk. Co-localization with glial cells was studied in aged xCT^+/+^ mice using double labeling with markers for astrocytes (anti-EAAT1/GLAST, **E**) and microglia (anti-Iba-1, **F**). Total xCT expression in the hippocampus of adult and aged xCT^+/+^ mice was analysed using Western blot analysis (**G**, n= 6 mice/group). System x_c_^-^ activity was determined by measuring [^3^H]-L-glutamate uptake (**H**, n=6-7 mice per group) in hippocampal synaptosomes. Data are presented as mean ± SEM and analyzed using a Mann-Whitney test, comparing adult and aged xCT^+/+^ mice. Abbreviations: GrDG, granular cell layer of dentate gyrus; LMol, lacunosum molecular layer; Mol, molecular layer of dentate gyrus; O, stratum oriens; P, pyramidal cell layer; R, stratum radiatum; v, blood vessel. Scale bar: 100μm (**A-D**), 20μm (**E,** **F**).

Hippocampal cysteine, GSH and GSSG levels are not affected by age or absence of system x_c_^-^, while lactate and pyruvate levels change with age

Untargeted metabolomics analysis of the hippocampus showed that neither cysteine levels, nor levels of the GSSG/GSH redox couple were altered by age or genotype (Supplementary Figure 7A-D). Age, but not the genotype affected the hippocampal lactate and pyruvate levels, suggesting metabolic alterations that might imply the presence of reductive stress in the hippocampus of aged mice (Supplementary Figure 7E-G).


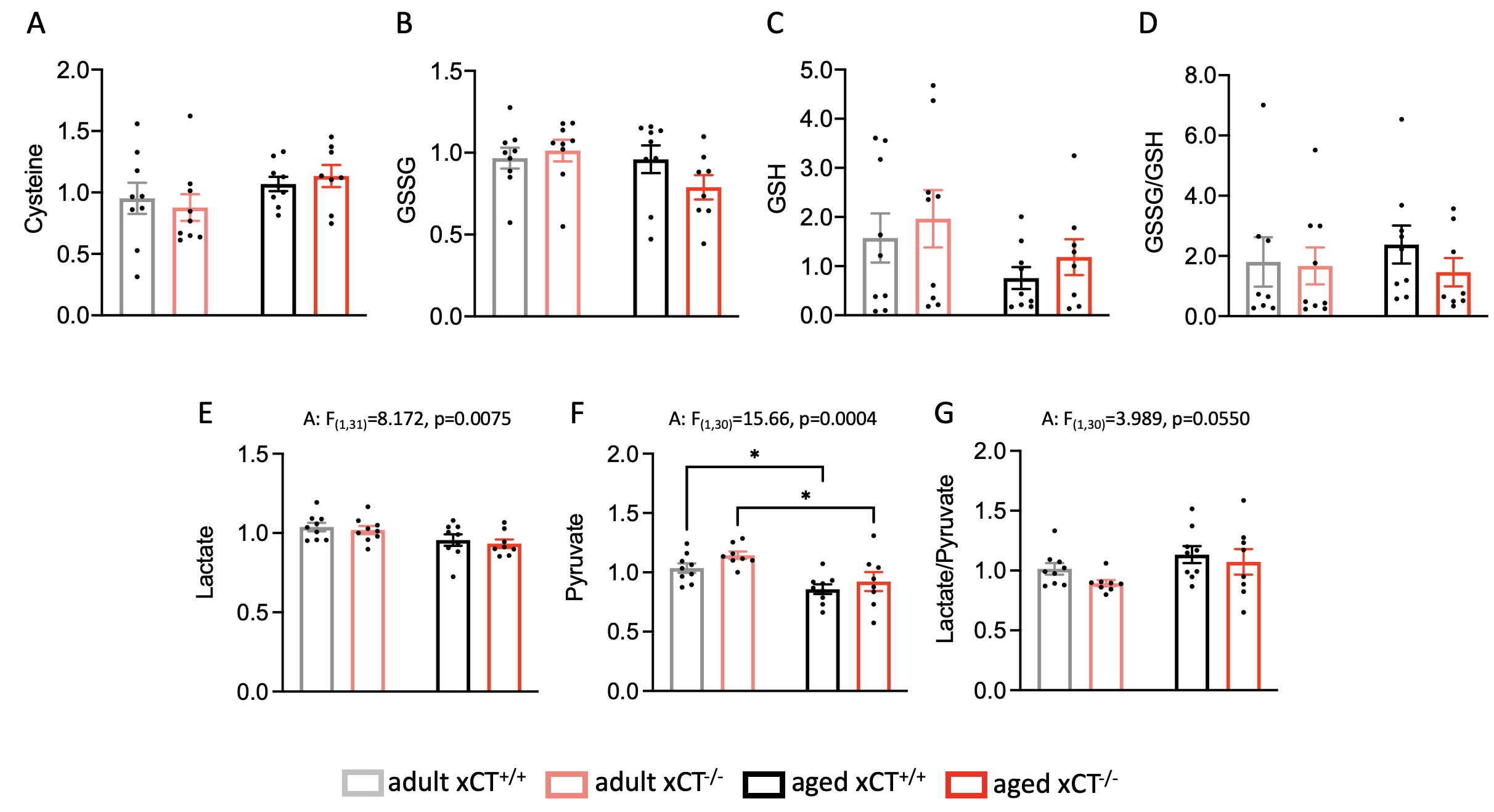


Supplementary Figure 7: Absence of xCT does not induce a shift in cysteine levels or in the GSSG/GSH redox couple in the hippocampus, but aging tends to increase the lactate/pyruvate ratio. Untargeted metabolomics of the hippocampus could detect cysteine (A), GSSG (B), GSH (C), lactate (E) and pyruvate (F). These levels were used to calculate the hippocampal GSSG/GSH (D) and lactate/pyruvate ratio (G), respectively (n=8-9 mice/group). Data are represented as mean scaled and imputed data ± SEM and analyzed using a two-way ANOVA, followed by Sidak's multiple comparisons test (see Supplementary Table 4). *p<0.05, A: aging effect.

Dendritic arborization of CA1 pyramidal neurons of aged xCT^-/-^ mice cannot be distinguished from adult xCT^+/+^ mice

A post-hoc analysis was performed for the one-by-one comparison of the Sholl analysis profiles of the basal (Supplementary Figure 8A-C) and the apical (Supplementary Figure 8D-F) tree was performed to further evaluate the genotype differences that are reported in Fig. 4 of the main text (for aging effects, see Fig. 4A-G of main text). In the basal tree, the number of intersections was decreased in the adult xCT^-/-^ mice, compared to the xCT^+/+^ mice (Supplementary Figure 8A). An opposite trend was seen in the aged mice (Supplementary Figure 8B), resulting in an overlap of the Sholl analysis profiles of the aged xCT^-/-^ and the adult xCT^+/+^ mice (Supplementary Figure 8C). No genotype differences were observed in the apical tree of the CA1 pyramidal neurons (Supplementary Figure 8D-F).


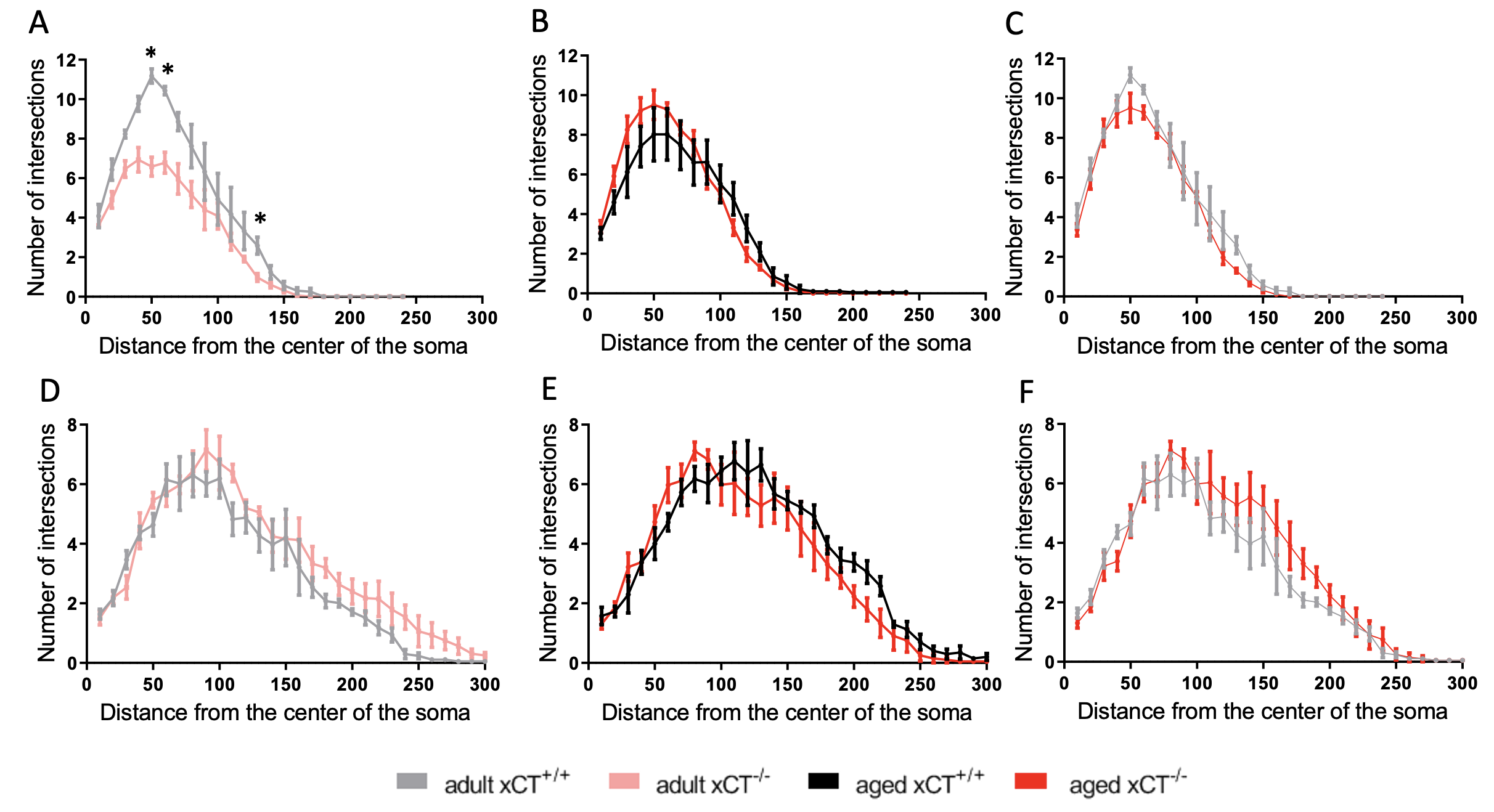


Supplementary Figure 8: Dendritic arborization of CA1 pyramidal neurons of aged xCT^-/-^ mice cannot be distinguished from adult xCT^+/+^ mice. Sholl analysis of the basal (A-C) and apical (D-F) tree was performed on a 2D tracing of the Golgi-Cox stained CA1 pyramidal neurons of adult and aged xCT^+/+^ and xCT^-/-^ mice. Three-five CA1 pyramidal neurons with clearly visible Golgi-Cox staining were selected per animal (n=4 mice/group), resulting in a total of 17-18 neurons/group. Data are presented as mean ± SEM and analyzed using a two-way ANOVA followed by Sidak's multiple comparisons test or a Kruskal-Wallis test in case of non-normal distributed data (A-C, distance 60, 160 and 170) (see Supplementary Table 4): ^*^p<0.05. In this figure multiple comparisons are shown; main effects are presented in Fig. 4 of the main text.

Supplementary tables

**Supplementary Table 1:** Overview of the number of mice used per experiment and distribution of the different cohorts per experimental setup

|  | **Adult** | | **Aged** | |
| --- | --- | --- | --- | --- |
|  | **xCT^+/+^** | **xCT^-/-^** | **xCT^+/+^** | **xCT^-/-^** |
| Lifespan |  |  | 42 | 41 |
| Cohort for health status and tissue analysis  Clinical frailty, body weight and temperature  Organ weight  Glucose  Western blotting/immunohistochemistry | 9  (9)  (8)  (8)  (6) | 12  (12)  (8)  (8)  (6) | 11  (7)  (11)  (8)  (6) | 10  (9)  (10)  (8)  (6) |
| Hippocampal cytokines | 7 | 5 | 7 | 7 |
| Age-induced priming of the immune system  Plasma cytokines | 11  (5) | 11  (5) | 13  (5) | 18  (5) |
| Immunofluorescence |  |  | 2 | 1 |
| Golgi-Cox staining | 4 | 4 | 4 | 4 |
| Barnes maze  Dynapenia  Slice electrophysiology  Plasma cystine/cysteine, GSH/GSSG  Sarcopenia and flow cytometry | 11  (10)  (7) | 11  (10)  (9) | 21  (12)  (6)  (10)  (9) | 17  (10)  (9)  (8)  (8) |
| Plasma cystine/cysteine, GSH/GSSG  Sarcopenia and flow cytometry | 8  (8) | 8  (6) |  |  |
| Cohort for clinical chemistry and metabolomics  Clinical chemistry  Metabolomics | 9  (9)  (9) | 9  (6)  (9) | 10  (8)  (9) | 14  (14)  (8) |
| Plasma pyruvate/lactate | 10 | 11 | 9 | 10 |
| Glutamate uptake | 6 | 3 | 7 |  |
| **Total** | **75** | **74** | **126** | **122** |

A total of 397 male mice were used in the current study. The numbers between brackets represent subgroups from the larger cohorts within the same table row.

Supplementary Table 2. Antibodies used for flow cytometry (A), western blotting, immunofluorescence and immunohistochemistry (B)

**A.**

| Cell surface marker | Clone | Catalogue number | Fluorochrome | Concentration |
| --- | --- | --- | --- | --- |
| **T-cell markers** | | | | |
| CD3 | 145-2C11 | 553062 | FITC | 3.125µg/mL |
| CD4 | RM4-5 | 550954 | PerCP-Cy 5.5 | 0.3125µg/mL |
| CD8 | 53-6.7 | 563068 | BV510 | 1.25µg/mL |
| CD62L | MEL-14 | 562404 | PE-CF594 | 0.625µg/mL |
| CD44 | IM7 | 561860 | PE | 0.625µg/mL |
| **Myeloid cells** | | | | |
| F4/80 | T45-2342 | 565411 | BV421 | 0.625µg/mL |
| CD11b | M1/70 | 550993 | PerCP-Cy 5.5 | 0.2µg/mL |
| CD11c | HL3 | 117317 | PE-Cy 7 | 1µg/mL |
| Ly6G | 1A8 | 561236 | Alexa Fluor 700 | 1.25µg/mL |
| **NK cells** | | | | |
| CD3 | 145-2C11 | 553062 | FITC | 3.125µg/mL |
| NKp46 | 29A1.4 | 560755 | Alexa Fluor 647 | 2.5µg/mL |
| CD160 | CNX46-3 | 562512 | PE CF594 | 10µg/mL |

**B.**

|  | Manufacturer | Catalogue No. | Concentration |
| --- | --- | --- | --- |
| **Primary antibodies** | | | |
| **Western blotting** | | | |
| Rabbit anti‐xCT | In house [9] |  | 0.13µg/mL |
| **Immunofluorescence** | | | |
| Rabbit anti-xCT | In house [3] | Ab#618 | 1µg/mL |
| Sheep-anti-EAAT1 | In house [10] | Ab#286 | 0.5µg/mL |
| Chicken-anti-Iba-1 | Synaptic System | 234006 | 2µg/mL |
| **Immunohistochemistry** | | | |
| Rabbit anti-mouse Iba-1 | Wako Pure Chemicals | 019-19741 | 1µg/mL |
| Rabbit anti-mouse GFAP | Dako | z0334 | 3.2µg/mL |
| **Secondary antibodies** | | | |
| **Western blotting** | | | |
| Horseradish peroxidase‐conjugated anti‐rabbit IgG | Dako | Po448 | 0.0318µg/mL |
| **Immunofluorescence** | | | |
| Alexa Fluor 488 Donkey Anti-Rabbit IgG (H+L) | ThermoFisher Scientific | A21206 | 2 µg/mL |
| Goat Anti-Chicken IgY (H+L) | ThermoFisher Scientific | A11039 | 2 µg/mL |
| Alexa Fluor 555 Goat Anti-Rabbit IgG (H+L) | ThermoFisher Scientific | A21429 | 2 µg/mL |
| Alexa Fluor 555 Donkey Anti-Sheep IgG (H+L) | ThermoFisher Scientific | A21436 | 2 µg/mL |

Antibodies for flow cytometry were purchased from BD biosciences and diluted in 30µl of FACS buffer.

Supplementary Table 3. Primer assays used for real-time PCR

| Gene | Assay ID |
| --- | --- |
| **Hippocampal inflammation** | |
| TNF-α | Mm00443258_m1 |
| IL-1β | Mm00434228_m1 |
| IL-6 | Mm00446190_m1 |
| Chil3 | Mm00657889_mH |
| **Age-induced priming** | |
| H2-DMa (MHC class II) | Mm00439226_m1 |
| **Reference genes** | |
| Brap | Mm00518493_m1 |
| Bcl2113 | Mm00463355_m1 |
| Ywhaz | Mm03950126_s1 |

All primer assays were purchased from ThermoFisher Scientific.

**Supplementary Table 4.** Overview of the statistical checks, data transformations and removed outliers throughout the manuscript

| **Figure** | **Normality (D'Agostino-Pearson)** | **Equal variances  (Browne-Forsythe)** | **Transformation** | **Post- transformation normality  (D'Agostino-Pearson)** | **Post- transformation equal variances  (Browne-Forsythe)** | **Results 2-way ANOVA post-transformation** | **Non-parametric 1-way ANOVA on original data** | **Outliers**  **(Grubbs' test,  α= 0.05)** |
| --- | --- | --- | --- | --- | --- | --- | --- | --- |
| 1C | No | No | Log | Yes | Yes | see fig. 1C | NA | aged WT:1 aged KO:1 |
| 1D | No | Yes | Log | Yes | Yes | see fig. 1D | NA | aged WT:1 aged KO:1 |
| 1E | No | No | Log | Yes | Yes | see fig. 1E | NA | aged WT:1 aged KO:1 |
| 1F | No | No | Log | Yes | Yes | see fig. 1F | NA | NA |
| 2D | Yes | No | NA | NA | NA | NA | NA | NA |
| 2E | No | Yes | NA | NA | NA | NA | no effect | NA |
| 2F | No | Yes | Log | Yes | Yes | see fig. 2F | NA | NA |
| 2H | Yes | Yes | NA | NA | NA | NA | NA | aged WT:1 |
| 2I | Yes | Yes | NA | NA | NA | NA | NA | adult WT:1; adult KO:1 |
| 2Q | Yes | Yes | NA | NA | NA | NA | NA | aged WT:1; aged KO:1 |
| 2R | No | No | Log | Yes | Yes | see fig. 2R | NA | adult WT:1; aged WT:1 |
| 2T | No | Yes | Log | Yes | Yes | see fig. 2T | NA | aged KO: 1 |
| 3I | NA | NA | Log2 | No | NA | NA | see fig. 3I | NA |
| 3K | NA | NA | Log2 | No | NA | NA | see fig. 3K | NA |
| 3M | NA | NA | Log2 | No | NA | NA | see fig. 3M | NA |
| 3P | NA | NA | Log2 | Yes | NA | NA | NA | 1 metabolite |
| 3R | Yes | Yes | NA | NA | NA | NA | NA | aged WT:1 |
| 3S | No | No | Log | Yes | No | see fig. 3S | NA | NA |
| 3T | Yes | Yes | NA | NA | NA | NA | NA | aged KO:1 |
| 4A-C basal tree - distance 60 | No | Yes | Log | No | Yes | NA | see fig. 4A | NA |
| Distance 100 | Yes | No | Log | Yes | Yes | no effect | NA | NA |
| Distance 110 | Yes | No | Log | Yes | No | NA | NA | NA |
| Distance 160 | No | Yes | 0 values | NA | NA | NA | no effect | NA |
| Distance 170 | No | Yes | 0 values | NA | NA | NA | no effect | NA |
| 4E-G apical tree - distance 120 | Yes | No | Log | Yes | No | NA | NA | NA |
| 4I | Yes | No | cube root | Yes | Yes | see fig. 4I | NA | NA |
| 4M-2Volt | No | Yes | Log | Yes | Yes | no effect | NA | NA |
| 4M-3Volt | No | Yes | Log | Yes | Yes | no effect | NA | NA |
| 4M-4Volt | No | Yes | Log | Yes | Yes | no effect | NA | adult WT:1 |
| 4M-5Volt | No | Yes | Log | Yes | Yes | no effect | NA | adult KO:1 |
| 4M-6Volt | No | Yes | Log | Yes | Yes | no effect | NA | NA |
| 4M-7Volt | No | Yes | Log | Yes | Yes | see fig. 4M | NA | NA |
| 4M-8Volt | No | Yes | Log | Yes | Yes | see fig. 4M | NA | NA |
| 4M-9Volt | No | Yes | Log | Yes | Yes | see fig. 4M | NA | NA |
| 4M-10Volt | No | Yes | Log | Yes | Yes | see fig. 4M | NA | NA |
| 4Q | Yes | Yes | NA | NA | NA | NA | NA | aged KO:1 |
| 4R | Yes | No | Log | Yes | No | NA | NA | NA |
| 5C | No | No | Log | Yes | Yes | see fig. 5C | NA | adult WT:1 |
| 5D | No | No | Log | No | Yes | NA | see fig. 5D | adult WT:1; aged WT:1; aged KO:1 |
| 5F | No | Yes | Log | Yes | Yes | see fig. 5F | NA | NA |
| 5G | No | Yes | Log | Yes | Yes | no effect | NA | NA |
| S2A | No | Yes | Log | Yes | Yes | see fig. S2A | NA | adult KO:1 |
| S2C | Yes | Yes | NA | NA | NA | NA | NA | adult KO:1 |
| S2F | Yes | No | Log | Yes | Yes | see fig. S2F | NA | aged KO:1 |
| S2J | Yes | No | Log | Yes | No | NA | no effect | NA |
| S2K | No | Yes | Log | Yes | Yes | no effect | NA | NA |
| S3B | Yes | No | Log | Yes | Yes | see fig. S3B | NA | adult KO:1 |
| S3E | Yes | No | Log | Yes | No | NA | NA | adult KO:1 |
| S3G | Yes | No | Log | Yes | No | NA | NA | aged WT:1 |
| S3I | No | Yes | Log | Yes | Yes | see fig. S3I | NA | adult KO:1: aged WT:1 |
| S3J | Yes | No | Log | Yes | Yes | see fig. S3J | NA | NA |
| S3K | No | No | Log | No | No | NA | no effect | NA |
| S3L | No | Yes | Log | Yes | Yes | see fig. S3L | NA | adult WT:1; aged KO:1 |
| S3M | Yes | Yes | NA | NA | NA | NA | NA | aged KO:1 |
| S3P | No | Yes | 0 values | NA | NA | NA | no effect | NA |
| S3R | Yes | No | Log | Yes | Yes | see fig. S3R | NA | aged KO:1 |
| S4B | No | No | Log | Yes | No | no effect | NA | NA |
| S4C | No | Yes | Log | No | Yes | NA | no effect | NA |
| S4F | Yes | No | Log | Yes | Yes | see fig. S4F | NA | NA |
| S4G | No | Yes | Log | Yes | Yes | see fig. S4G | NA | NA |
| S4H | Yes | No | Log | Yes | Yes | see fig. S4H | NA | NA |
| S4L | No | No | Log | Yes | Yes | see fig. S4L | NA | NA |
| S4M | Yes | Yes | NA | NA | NA | NA | NA | adult WT:1; adult KO:1; aged KO:1 |
| S4N | Yes | Yes | NA | NA | NA | NA | NA | aged WT:1; aged KO:1 |
| S4P | Yes | No | Log | Yes | Yes | see fig. S4P | NA | aged WT:1 |
| S7D | No | Yes | Box-cox  (λ=-1) | Yes | Yes | no effect | NA | adult WT:1 |
| S7F | Yes | Yes | NA | NA | NA | NA | NA | adult KO:1 |
| S7G | Yes | No | Log | Yes | Yes | see fig. S7G | NA | adult KO:1 |
| S8A | see figure 4A-C | | | | | | | |
| S8B | see figure 4A-C | | | | | | | |
| S8C | see figure 4A-C | | | | | | | |
| S8D | see figure 4E-G | | | | | | | |
| S8E | see figure 4E-G | | | | | | | |
| S8F | see figure 4E-G | | | | | | | |

Figures not mentioned in the table contain data that meet all the assumptions of the respective statistical test performed and no outliers were removed from the dataset.

**References**

1. Ottestad-Hansen S, Hu QX, Follin-Arbelet VV, Bentea E, Sato H, Massie A, et al. The cystine-glutamate exchanger (xCT, Slc7a11) is expressed in significant concentrations in a subpopulation of astrocytes in the mouse brain. Glia. 2018;66:951–970.

2. Albertini G, Deneyer L, Ottestad-Hansen S, Zhou Y, Ates G, Walrave L, et al. Genetic deletion of xCT attenuates peripheral and central inflammation and mitigates LPS-induced sickness and depressive-like behavior in mice. Glia. 2018;66:1845–1861.

3. Van Liefferinge J, Bentea E, Demuyser T, Albertini G, Follin-Arbelet V, Holmseth S, et al. Comparative analysis of antibodies to xCT (Slc7a11): Forewarned is forearmed. J Comp Neurol. 2016;524:1015–1032.

4. Garcia ADR, Doan NB, Imura T, Bush TG, Sofroniew M V. GFAP-expressing progenitors are the principal source of constitutive neurogenesis in adult mouse forebrain. Nat Neurosci. 2004;7:1233–1241.

5. Santhanam P, Khitan Z, Khthir R. Association between serum total bilirubin and serum creatinine and the effect of hypertension. J Clin Hypertens (Greenwich). 2015;17:61–62.

6. Regino WO, Velasco H, Sandoval H. The protective role of bilirubin in human beings. Rev Col Gastroenterol. 2009;24:293–301.

7. Salam N, Rane S, Das R, Faulkner M, Gund R, Kandpal U, et al. T cell ageing: effects of age on development, survival &amp; function. Indian J Med Res. 2013;138:595–608.

8. Loukov D, Naidoo A, Puchta A, Marin JLA, Bowdish DME. Tumor necrosis factor drives increased splenic monopoiesis in old mice. J Leukoc Biol. 2016;100:121–129.

9. Massie A, Schallier A, Mertens B, Vermoesen K, Bannai S, Sato H, et al. Time-dependent changes in striatal xCT protein expression in hemi-Parkinson rats. Neuroreport. 2008;19:1589–1592.

10. Li Y, Zhou Y, Danbolt NC. The rates of postmortem proteolysis of glutamate transporters differ dramatically between cells and between transporter subtypes. J Histochem Cytochem. 2012;60:811–821.
